# Group B Streptococcus Interactions with Human Meningeal Cells and Astrocytes *In Vitro*

**DOI:** 10.1371/journal.pone.0042660

**Published:** 2012-08-10

**Authors:** Khalil Alkuwaity, Alexander Taylor, John E. Heckels, Kelly S. Doran, Myron Christodoulides

**Affiliations:** 1 Neisseria Research Group, Molecular Microbiology, Clinical and Experimental Sciences, Sir Henry Wellcome Laboratories, University of Southampton Faculty of Medicine, Southampton General Hospital, Southampton, United Kingdom; 2 Department of Biology, San Diego State University, San Diego, California, United States of America; Columbia University, United States of America

## Abstract

**Background:**

*Streptococcus agalactiae* (Group B Streptococcus, GBS) is a leading cause of life-threatening neonatal meningitis and survivors often suffer permanent neurological damage. How this organism interacts with the meninges and subsequently with astrocytes that constitute the underlying cortical *glia limitans superficialis* is not known.

**Methodology/Principal Findings:**

In this paper, we demonstrate dose-dependent adherence of GBS over time to human meningioma cells and fetal astrocytes *in vitro*, which was not influenced by expression of either β-haemolysin/cytolysin (β-h/c) toxin, different capsule serotypes or by absence of capsule (p>0.05). Internalization of GBS by both cell types was, however, a slow and an infrequent event (only 0.02–0.4% of associated bacteria were internalised by 9 h). Expression of β-h/c toxin did not play a role in invasion (p>0.05), whereas capsule expression lead to a reduction (p<0.05) in the numbers of intracellular bacteria recovered. GBS strains induced cytotoxicity as demonstrated by the measurement of lactate dehydrogenase (LDH) enzyme release by 9 h and by viable staining. Increasing levels of meningioma cell death correlated with bacterial growth and the phenotype of β-h/c toxin production, *i.e.* from weakly, to normo- to hyper-haemolytic. However, cytotoxicity was significantly greater (p<0.05) towards astrocytes, and infection with initial MOI≥0.003 induced 70–100% LDH release. By comparing wild-type (β-h/c^+^) and mutant (*ΔcylE* β-h/c^−^) strains and β-h/c toxin extracts and by using the surfactant dipalmitoylphosphatidylcholine in cytotoxicity inhibition experiments, β-h/c toxin was demonstrated as principally responsible for cell death.

**Conclusions/Significance:**

This study has described key events in the interactions of GBS with meningeal cells and astrocytes *in vitro* and a major virulence role for β-h/c toxin. Understanding the mechanisms involved will help to identify potential therapies for improving patient survival and for reducing the incidence and severity of neurological sequelae.

## Introduction

*Streptococcus agalactiae* (Group B Streptococcus, GBS) is a leading cause of life-threatening neonatal infections that include pneumonia, sepsis and meningitis. Recent estimates of the rates of GBS meningitis in neonates in the US and in the UK is 0.65 and 0.72 per 1,000 live births respectively [1], [2] and can occur as early-onset disease (EOD) or late-onset disease (LOD). EOD occurs principally in infants aged 0–7 days and in 80% of cases the initial manifestation is respiratory distress [3], with progression to pneumonia, septicaemia and meningitis in 9%, 83% and 7% of cases, respectively [4]. LOD principally occurs in neonates 7 days to 3 months of age and initial presentation includes fever, lethargy and tachypnoea, and sepsis and meningitis in 65% and 27% of cases, respectively [3], [4]. GBS are grouped into 9 serotypes (Ia, Ib, II-VIII) based on antigenic differences in the structure of the capsular polysaccharide and serotypes Ia, III and V have been reported to account for about 80% and 92% of EOD and LOD cases, respectively [3]. Disease mortality has decreased over the last four decades, from 55% in the 1970s and 10% in the 1980s to 4–6% from 1990–2005 [4], [5] and this has been attributed to intrapartum antibiotic prophylaxis. However, between 36–50% of survivors of GBS meningitis will suffer permanent neurological sequelae, hearing loss, seizures and mental retardation [6].

Although significant advances have been made in understanding the pathophysiology of GBS infection and the roles of specific bacterial virulence factors [7], [8], the nature of GBS interactions with the human meninges is unknown at the cellular and molecular levels. In man, the meninges comprise the pachymenix or dura mater, and the leptomeninges, which provide the largest surface area of cells within the subarachnoid space (SAS) and consist of the *arachnoid mater* and *pia mater* together with the trabeculae that traverse the cerebrospinal fluid (CSF)-filled SAS [9]–[11]. The *pia mater* closely follows the contours of the human brain and enters sulci and is separated by a sub-pial space from the *glia limitans superficialis*, which surrounds the entire surface of the brain and spinal cord and is composed of compacted astrocytes [12]. A likely portal of entry of GBS into the SAS is penetration of the blood-cerebrospinal fluid (B-CSF) barrier of blood vessels in the SAS and an *in vitro* model of brain microvascular endothelial cells (BMEC) is being extensively used to examine how GBS ligand-host cell receptor interactions enable endothelial penetration [13]–[21]. However, the subsequent interactions of GBS with cells of the meninges have not been examined.

Animal models such as the rat and mouse have provided much valuable information on the pathogenesis of bacterial meningitis, but they do have their limitations [22]. Moreover, there are anatomical differences in the membranes and SAS of experimental animals compared to humans; for example, arachnoid trabeculae are absent in the mouse leptomeninx [23], the SAS is restricted in rodents [24] and zonula adhaerens are present between rat arachnoid cells whereas desmosomal junctions are found in humans [25]. In addition, for surrogate cell culture models, primary human leptomeningeal cells cannot be cultured reliably *in vitro* [26]. Thus, we established an *in vitro* model of the leptomeninges to study bacterial infection, using cells cultured from meningiomas, which are benign tumours that arise from the leptomeninges. Meningioma cells have the same cytological and morphological structure as cells throughout the leptomeninges [9], [26], [27] and importantly, meningeal bacterial pathogens show similar patterns of interaction with fresh leptomeninges and meningioma cells [28]. An important role for the *pia mater* meninges is to provide a physical and physiological barrier that prevents solutes reaching the underlying brain [29]. Meningioma cells are suitable for studying the barrier functions of the meninges *in vitro* and of the *pia mater* in particular [26]. The model has been used to demonstrate that important meningeal pathogens (*Neisseria meningitidis*, *Haemophilus influenzae*, *Streptococcus pneumoniae* and *Escherichia coli* K1) interact differently with meningeal cells *in vitro*, with respect to their ability to adhere to and invade host cells and to induce inflammation and cell death [30], [31]. In the current study, we tested *in vitro* the hypothesis that GBS infection compromises the barrier properties of cells derived from the human meninges to allow bacterial infection to involve underlying astrocyte cells, which constitute the *glia limitans superficialis.*

## Results

### GBS adhere to human meningioma cells

**Figure 1:**
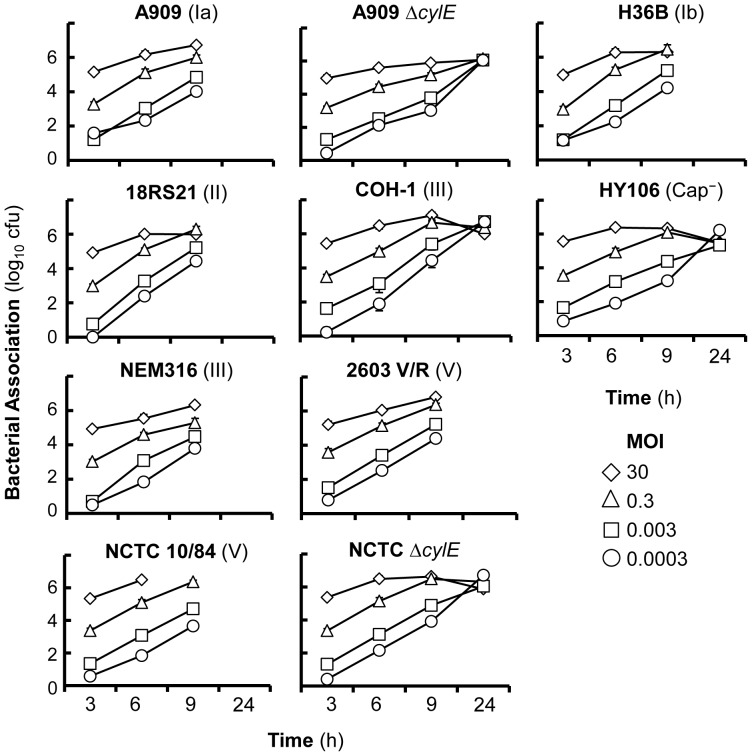
Association of GBS strains with human meningioma cell lines. Human meningioma cell lines were infected with various MOI (0.0003–3000) of GBS strains of different serotypes and also with strains deficient in expression of β-h/c toxin (Δ*cylE*) or capsule. Bacterial association was quantified over time with the symbols representing the mean and the error bars the standard error of the mean from three independent experiments. Similar data were obtained using both cell lines.

Human meningioma cells were challenged with various concentrations of each GBS serotype (Table 1) and bacterial adherence quantified. GBS bacteria demonstrated a dose-dependent association with cell monolayers (Figure 1) and in general, no significant differences were observed between the 7 wild type capsulate strains for association at any of the doses tested over time (*P*>0.05). However, association following infection with the highest MOI (3000) of wild-type bacteria tested could not be quantified at 3 h, as visual inspection showed destruction and sloughing off of the meningioma cell monolayers from their collagen basements. Infection with an initial MOI of 30 led to a rapid saturation of the monolayers (Figure 1) followed by monolayer destruction after 9 h. By contrast, all monolayers were visually intact at 9 h after infection with initial MOI of between 0.0003–0.3 of all wild type bacteria tested, but with damage of cell monolayers occurring by 24 h post infection. The hyper-haemolytic NCTC 10/84 strain (serotype V) was significantly cytotoxic, since unlike the other wild type strains, infection with an initial MOI of 30 resulted in destruction of cell monolayers between 6–9 h. By contrast, the weakly haemolytic COH-1 strain (serotype III) was the least cytotoxic, and regardless of the initial MOI used to infect cells, similar levels of adherent bacteria were recovered from intact monolayers at 24 h post infection (p>0.05)(Figure 1).

**Table 1:** Bacterial strains used in the study.

Organism	ATCC Reference No.	Capsular type	β-haemolysis	Comments	Reference
*S. agalactiae*					
A909	BAA 1138	Ia	++	WT	[68]
A909 Δ*cylE*	––	Ia	−	β-h/c deficient	[69]
H36B	BAA 1174	Ib	++	WT	[70]
18RS21	BAA 1175	II	++	WT	[70]
NEM 316	ATCC 12403	III	++	WT	[71]
2603V/R	BAA 611	V	++	WT	[70]
COH-1	BAA 1176	III	+	WT, Hyper-capsulated	[72]
COH-1 HY106	––	––	+	Uncapsulated	[73]
NCTC 10/84	ATCC 49447	V	+++	WT, Hyper-haemolytic	[74]
NCTC 10/84 Δ*cylE*	––	V	−	β-h/c deficient	[69]
*N. meningitidis* MC58	––	B	−	––	[60]
*E. coli* IH3080	––	O18:K1	−	––	[61]

WT, wild type; − Non-haemolytic; + Weakly haemolytic; ++ Normo-haemolytic; +++ Hyper-haemolytic.

There were no significant differences (p>0.05) in the dynamics of association between the Δ*cylE* β-haemolysin/cytolysin (β-h/c) toxin mutants and their parent strains by 9 h, but adherent mutant bacteria could be recovered at 24 h from intact monolayers. Also, there were no significant differences in the levels of association of the acapsulate HY106 strain, compared to the parent COH-1 strain (*P*>0.05) (Figure 1).

Control experiments were also done to demonstrate that adherence of GBS to plastic surfaces was irrelevant. GBS strains were grown in cell culture wells without cell monolayers and as expected, adherence to plastic surfaces was negligible and was <0.5% of the bacterial growth in a well at any of the time points sampled during growth (Figure S1A). The relative growth rates of the wild-type GBS strains in culture medium over time were also examined. All of the wild-type strains exhibited similar growth curves (P>0.05). In addition, there were no differences statistically (P>0.05) in the growth rates between the wild-type strains and their isogenic mutants, deficient in either capsule or β-h/c toxin (Figure S1B).

Scanning electron microscopy analyses of infected cell monolayers (Figure S2A) confirmed the viable count data by demonstrating increases in the numbers of adherent bacteria over time. Few bacteria were visible on meningioma cell surfaces by 3–6 h, but by 9 h, large clusters and chains of adherent GBS dominated the host cell surfaces. By 24 h, total cell monolayer damage was induced by wild-type bacteria and only clusters of bacteria and cell debris were observed scattered on the surface of the Transwell insert membranes. By contrast, monolayers were intact at 24 h after infection with the weakly-haemolytic COH-1 strain and the β-h/c^−^ mutant strains (images not shown).

### Human meningioma cells provide an effective barrier to GBS invasion

Meningioma cell monolayers were infected with an initial MOI of 0.3 (10^4^ cfu) of the different GBS serotypes and cellular invasion was quantified using the gentamicin-cytochalasin D (CD) assay. Cellular invasion was not detectable at 3 h or 6 h for any of the wild type GBS serotypes (*P*>0.05). Although the levels of invasion by GBS strains increased to significance by 9 h, the numbers of recovered bacteria after gentamicin treatment as a percentage of associated bacteria were very low (≤0.4%) (Table 2). Nevertheless, the acapsulate strain HY106 did show a significantly higher rate of invasion (∼0.2%) than its capsulated parent strain COH-1(∼0.02%; P<0.05) (Table 2). There was no significant difference in invasion between the normo-haemolytic A909 (β-h/c^+^) wild-type strain and the A909Δ*cyl*E (β-h/c^−^) mutant (p>0.05). However, for the hyper-haemolytic strain NCTC 10/84, the percentage invasion rate (∼0.02%) was significantly lower (P<0.05) than that calculated for its corresponding *ΔcylE* variant (∼0.15%). It was possible that excessive pore-formation by this strain allowed the gentamicin to leak inside the cells and kill internalised bacteria, leading to reduced recovery of viable bacteria [13]. In order to test this hypothesis, meningioma cell monolayers (n = 3 experiments) were infected with an initial MOI of 0.3 of the hyper-haemolytic NCTC 10/84 strain and treated with the surfactant dipalmitoylphosphatidylcholine (DPPC; 3 mg/ml), which has been shown to inhibit β-h/c pore-formation. There was a significant (*P*<0.01) increase in the number of bacteria recovered after gentamicin treatment in the presence of DPPC, with the number of internalised bacteria increasing from 500 (±90) cfu/monolayer without DPPC to 3,700 (±540) cfu/monolayer with DPPC. This number of recovered bacteria was similar (P>0.05) to the levels observed for the NCTC10/84 Δ*cyl*E β-h/c^−^ strain (2400±500 cfu/monolayer; mean invasion rate of 0.149%, Table 2). In separate control experiments, we confirmed that the invasion rate of the NCTC10/84 Δ*cyl*E β-h/c^−^ strain was unaffected by DPPC (3 mg/ml): the invasion rate in the absence and presence of DPPC was 0.31% and 0.4% respectively (n = 2 independent experiments, P>0.05).

**Table 2:** Invasion of meningioma cells by GBS.

GBS strain	% Mean Invasion (± SEM)*
A909 (Ia)	0.328 (0.094)
A909 Δ*cylE*	0.413 (0.131)
H36B (Ib)	0.059 (0.033)
18RS21 (II)	0.051 (0.025)
NEM316 (III)	0.148 (0.066)
COH-1 (III)	0.015 (0.006)
HY106 (Ca 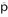 )	0.175 (0.089)
2603 V/R (V)	0.073 (0.025)
NCTC 10/84 (V)	0.019 (0.006)
NCTC 10/84 Δ*cylE*	0.149 (0.092)

Monolayers were infected for 9 h with an initial MOI of 0.3 (10^4^ cfu bacteria), washed and then treated with gentamicin.

* The percentage of invading bacteria (invasion rate) was calculated using the formula: Invasion rate (%) = (internalised bacteria/associated bacteria)×100. The mean and SEM for GBS were calculated from n = 3–6 experiments. *E. coli* IH3080 was included as a positive control and showed an invasion rate of 1.167% (SEM ± 0.283 from n = 10 experiments). Similar data were obtained using both meningioma cell lines.

Although there was a significant reduction of recovered bacteria (*P*<0.05) in all the monolayers treated with gentamicin and CD, compared to treatment with gentamicin alone for all the strains tested (Figure S3), GBS internalisation by meningioma cells was a rare event. Indeed, examination of a large number of grids by transmission electron microscopy only demonstrated the occasional intracellular bacterium (Figure S2B).

### GBS infection causes meningioma cell death

The release of LDH from meningioma cells was used to quantify cell death induced by infection with wild-type GBS strains, since measurement of LDH release has previously been shown to be a reliable biochemical indicator of cell injury in other *in vitro* cell culture models [32]. Preliminary experiments showed that GBS infection (with initial MOI from 0.03–3000) did not induce LDH release at 3 h and 6 h (data not shown) and therefore measurements were taken at 9 h as association experiments suggested that cell death was beginning to occur by this time (Figure 1).

**Figure 2:**
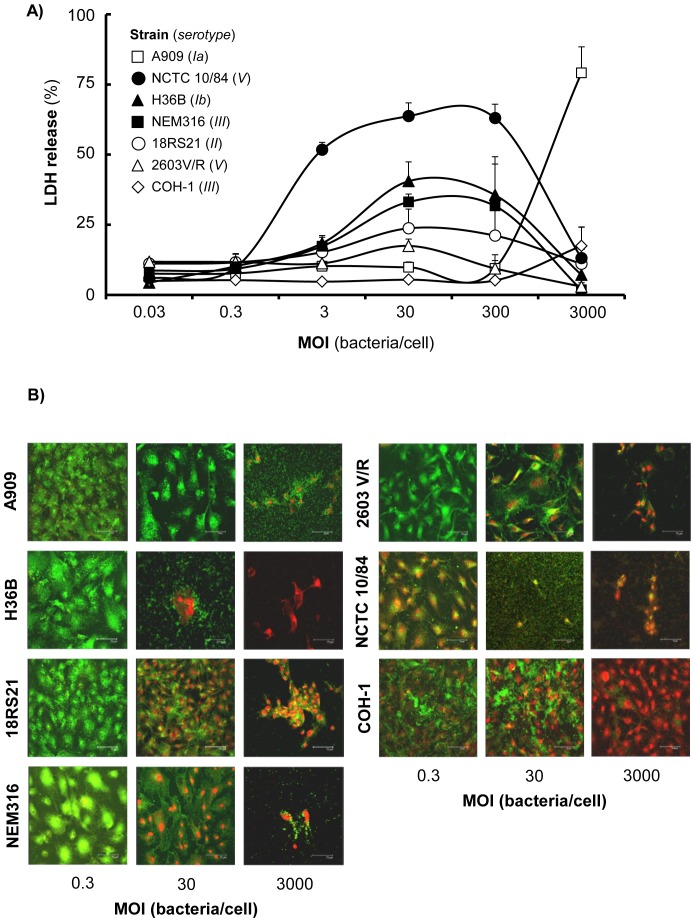
Meningioma cell injury induced by wild type GBS strains. A) Release of LDH: cells were infected for 9 h with different initial MOI of GBS strains and LDH release was measured. Results shown are the mean values of LDH release compared with maximum release by lysed cells and the error bars are the SEM from at least two independent experiments, each performed in triplicate. B) Confocal microscopy: cell cultures were infected with a range of initial MOI of different serotypes of wild type GBS for 9 h. Cell death was examined using the LIVE/DEAD fluorescent dye assay, where uptake of the red dye (ethidium homodimer) identifies dead cells and uptake of the green dye (calcein AM) identifies live cells. The scale bar shows 75 µm (×40 lens magnification). Images are of monolayers infected for 9 h, except for the COH-1 strain infected for 24 h, and they are representative of experiments carried out in triplicate. Similar data were obtained using both cell lines.

LDH release was detectable at 9 h after infection with initial MOIs≥3 of wild type GBS serotypes and significant differences were observed in the patterns of LDH release (Figure 2A). The most pronounced LDH release was induced by the hyper-haemolytic NCTC 10/84 (V) strain, where LDH release reached ∼50% after infection with initial MOI as low as 3, and about 60–70% after infection with initial MOIs of 30 and 300. Strains 18RS21 (II), 2603V/R (V), NEM316 (III) and H36B (1b) induced between ∼20–50% LDH release after infection with initial MOIs of 30 and 300 (Figure 2A). However, infection with an initial MOI of 3000 was needed for strain A909 (Ia) to induce LDH release by 9 h (70%). The lowest LDH release was observed with the weakly haemolytic COH-1 strain, where no LDH release above the baseline (average = 7.8%±0.64) was detected except after infection with an initial MOI of 3000, which induced ∼20% enzyme release. Regardless, complete damage and sloughing off from the collagen basement of the infected monolayers was observed at 24 h for all infecting doses for all wild-type strains, except for COH-1, and LDH release could not be detected.

A fluorescent dye-based LIVE/DEAD assay was also used to assess host cell viability and cytotoxicity. Meningioma cell monolayers were infected with initial MOI of 0.3, 30 and 3000 of each wild-type bacterial strain for up to 9 h, and for 24 h in the case of the weakly-haemolytic strain COH-1. The choice of these MOI was based on the observations made from LDH results so that varying degrees of cell death would be induced (Figure 2A). Examination by confocal microscopy demonstrated significant differences in dye interactions with meningioma cells following GBS infection (Figure 2B) and in general these observations correlated with the LDH assay data. With the lowest initial MOI of 0.3 tested, the strains did not induce cell death as the monolayers were intact and viable (stained green), except for NCTC 10/84, which induced red dye uptake indicative of cell death. With the higher initial MOI of 30, two patterns of cytotoxicity were observed: monolayers challenged with NCTC 10/84 and H36B were completely damaged with only a few dead (stained red) cells or cell fragments remaining. Monolayers challenged with other strains (18RS21, NEM316 and 2603V/R) were still intact but showed a mixture of green and red cell staining, suggesting that cell death was starting to occur, whereas monolayers challenged with the A909 strain remained viable. With the highest initial MOI of 3000, complete destruction of the monolayers was induced by all the strains, with only cellular debris remaining. By contrast, with the weakly haemolytic COH-1 strain, the challenged monolayers were completely intact at 9 and 24 h regardless of the initial MOI tested, but a gradual change in the staining reaction was apparent at 24 h, with red dye uptake increasing with bacterial dose. With the highest initial MOI of 3000, although all the cells within the monolayer were dead, there was no significant loss of monolayer integrity.

### The β-haemolysin/cytolysin (β-h/c) toxin plays a critical role in meningioma cell death

**Figure 3:**
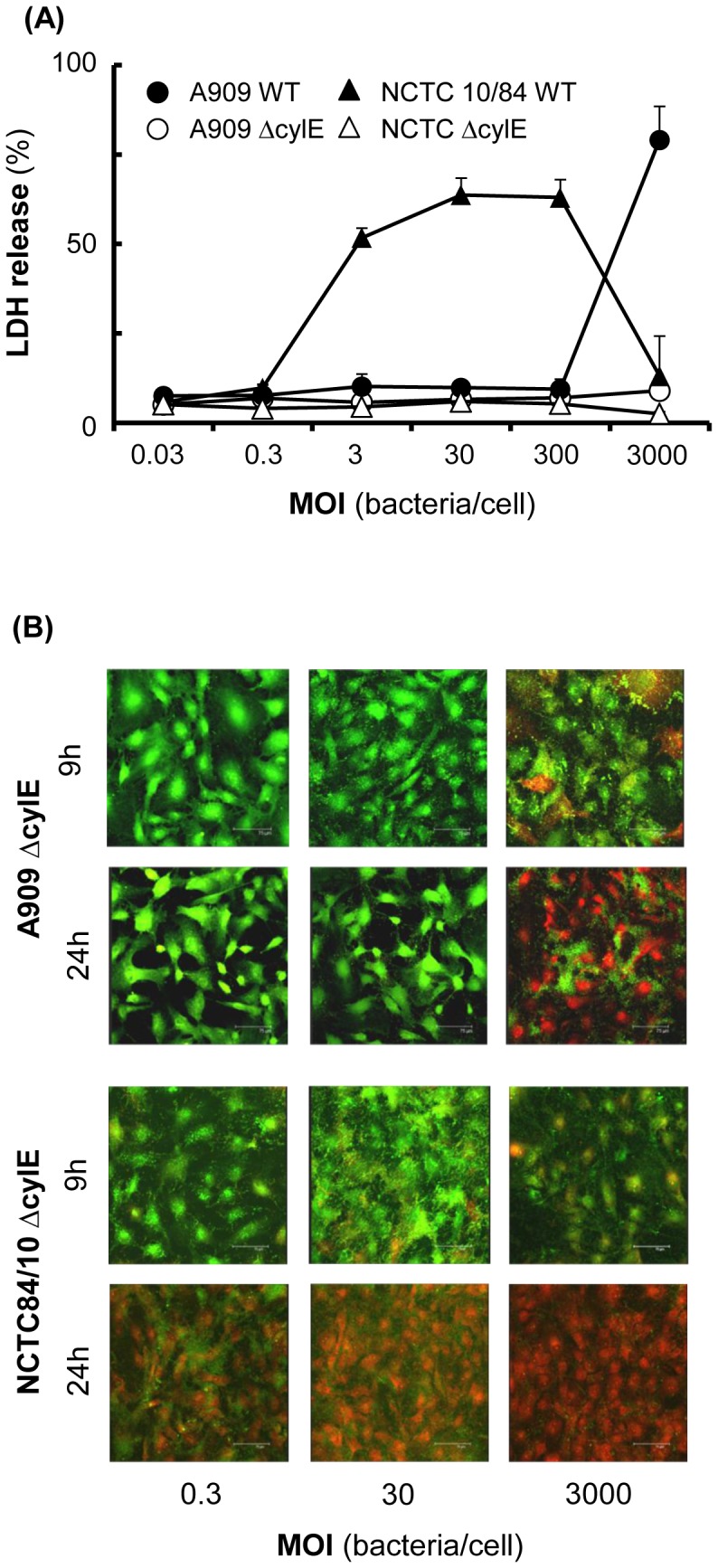
Comparison of β-hemolysin/cytolysin (β-h/c) toxin-expressing and toxin-deficient strains on meningioma cell death. Cell cultures were infected with various doses of wild-type A909 and NCTC10/84 (β-h/c^+^) strains and their isogenic Δ*cylE* (β-h/c^−^) mutants and meningioma cell death quantified after 9 h. (A) Bacterial dose-dependent LDH release from meningioma cells. The symbols represent the mean values of LDH release compared with maximum release by lysed cells and the error bars are the SEM from at least two independent experiments. (B) Confocal microscopy images of meningioma cells challenged with different concentrations of GBS A909 and NCTC10/84 Δ*cylE* mutant strains for 9 h and 24 h. The red colour identifies dead cells, whereas the green colour corresponds to viable cells. The scale bar at bottom right of each image measures 75 µm (×40 magnification). Similar data were obtained using both cell lines.

Next, we tested the hypothesis that meningioma cell death was due to the effects of β-h/c toxin produced by GBS, by infecting cell monolayers with the normo- and hyper-haemolytic parent strains A909 and NCTC 10/84 and their corresponding Δ*cylE* (β-h/c^−^) variants. Initially, measurements of LDH release were taken at 9 h post infection. The A909 strain induced approximately 75% LDH release after infection with an initial MOI of 3000, whereas the NCTC84/10 strain induced ≥60% cell death with initial MOI of ≥3 (Figure 3A). By contrast, the Δ*cylE* mutants did not induce any significant (P>0.05) LDH release above the base line with any MOI tested by 9 h (average of 6.4±0.8%) or 24 h (average 6.3±1.6%) (Figure 3A).

The LIVE/DEAD assay with confocal imaging confirmed that after 9 h of challenge, both of the β-h/c*^−^* mutant strains did not induce cell death after infection with initial MOI of 0.3 and 30. There was some induction of cell death after initial infection with a MOI of 3000, where the monolayers showed some uptake of the red dye, but the majority of the cells were stained green and the monolayers were completely intact (Figure 3B). By contrast, infection with both of the parent strains at an initial MOI of 3000 caused the complete destruction of monolayers (Figure 2B). Confocal imaging at 24 h showed completely intact monolayers, but different staining reactions were observed (Figure 3B). Cells within monolayers challenged with the A909 Δ*cylE* strain were completely viable (green stain) following infection with initial MOI of 0.3 and 30. However, after infection with an initial MOI of 3000, the monolayers were intact but the majority of cells were non-viable (red stain) (Figure 3B). Monolayers infected with the NCTC10/84 Δ*cylE* strain also remained intact but demonstrated a gradual increase in cell death after infection with different MOI and the effect was more pronounced than with the A909 Δ*cylE* strain (Figure 3B).

**Figure 4:**
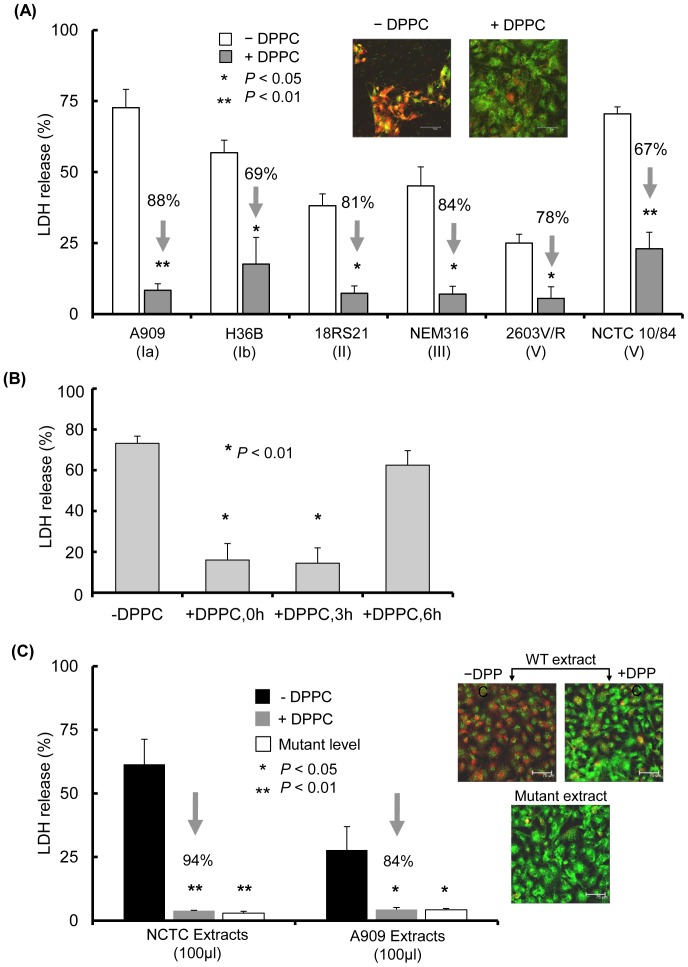
The surfactant DPPC protects meningioma cells from GBS-induced death. (A) Effect of DPPC on cell death induced by live bacterial infection. Meningioma cells were infected with doses of GBS strains that induced the highest levels of LDH release (Figure 2A) and cell death quantified at 9 h. The columns represent the mean levels of LDH released from monolayers and the arrows indicate the percentage of reduction of LDH release in the presence of DPPC in comparison with LDH in the absence of DPPC. The error bars show the SEM of at least two independent experiments. The two confocal microscopy images show monolayers infected with a cytotoxic dose (initial MOI, 30) of the hyper-haemolytic NCTC10/84 strain for 9 h in the absence and presence of DPPC. (B) The protective effect of DPPC is dependent on time of addition. Meningioma cell monolayers were infected with GBS strain NCTC10/84 and DPPC (3 mg/ml) added at 0, 3 and 6 h. LDH release was measured at 9 h. The columns represent the mean levels of LDH release and the error bars the SEM of 3 independent infection experiments. (C) Cell death induced by β-h/c toxin extracts and inhibition by DPPC. The graph shows LDH release from monolayers treated with β-h/c toxin extracts prepared from wild type NCTC and A909 strains (containing 100HU and 50HU, respectively). Equal volumes of extracts prepared from their β-h/c deficient mutant strains were also used. Results shown are mean values of LDH release and error bars are the SEM from four independent experiments using two independent batches of β-h/c toxin extracts. Arrows indicate the percentage inhibition of LDH release in the presence and absence of DPPC. The confocal microscopy images show monolayers treated with β-h/c extracts (250HU) prepared from wild type GBS strains for 9 h in the absence and presence of DPPC. An equivalent volume of extract prepared from the Δ*cylE* (β-h/c deficient) mutant strain was also used. Using the LIVE/DEAD assay, the red colour identifies dead cells, whereas the green colour corresponds to viable cells. The scale bar at bottom right of each image measures 75 µm (×40 magnification). Similar data were obtained using both cell lines.

The protective effect of DPPC against cell injury induced by GBS strains was investigated next. Pilot dose-finding experiments demonstrated that a concentration of 3 mg/ml of DPPC was optimal for inhibiting LDH release induced by GBS infection (Figure S4A). For the LDH release experiments, MOI were chosen that had been shown to induce the highest levels of LDH release from meningioma cells (Figure 2A), ranging from 30–3000 depending on the strain. In the presence of DPPC, LDH release induced by the GBS strains was significantly reduced (p<0.05) by ∼67–84% (Figure 4A). The inhibitory effects of DPPC against cell injury were confirmed using the fluorescent dye-based assay (Figure 4A inset). Monolayers challenged with NCTC 10/84 strain showed significant levels of cell death by 9 h, as demonstrated by the presence of the red dye within cells, cell fragments and cell nuclei. By contrast, in the presence of DPPC, cell monolayers were still intact and the majority of cells were viable (green stain) (Figure 4A inset).

Next, the protective effect of DPPC, when added at later time points, was investigated. Cell monolayers were infected with an initial MOI of 30 of strain NCTC10/84 for 9 h, which induced ∼75% LDH release (Figure 4B). Addition of DPPC (3 mg/ml) at both 0 h and 3 h reduced LDH release by ∼80% (p<0.01), but this inhibitory effect was lost if the surfactant was added at 6 h post-infection (p>0.05) (Figure 4B).

In order to substantiate the role of the β-h/c toxin in meningioma cell death, monolayers were treated with crude haemolysin extracts prepared from the wild type strains A909 and NCTC 10/84 and their isogenic Δ*cylE* mutants and the release of cellular LDH was quantified at 9 h post infection. The extract (100 µl) prepared from the hyper-haemolytic NCTC strain contained 100HU and induced LDH release by approximately 60%, whereas an equal volume of extract prepared from the normo-haemolytic A909 strain, which contained 50HU induced approximately 30% LDH release (Figure 4C). By contrast, extracts prepared from both of the Δ*cylE* mutants did not induce any significant release of LDH above base-line (mean 4.1±0.9%). Furthermore, in the presence of DPPC (3 mg/ml), LDH release that was induced by extracts from both wild type strains was reduced by 85–95% (p<0.05), to levels that were similar to base-line and mutant levels (Figure 4C).

Meningioma cell monolayers were also treated for 9 h with equal volumes of crude extracts prepared from the wild type NCTC10/84 strain and its Δ*cylE* variant, in the presence (3 mg/ml) or absence of DPPC and subjected to the LIVE/DEAD assay with confocal imaging. A dose of extract prepared from the wild type GBS strain (containing 250HU, which was equivalent to 50HU in the LDH assay) induced cell injury as judged by the uptake of the red dye by the monolayer. By contrast, the extracts from the Δ*cylE* strain did not induce cell injury. Moreover, the addition of DPPC was protective, since the monolayers took up the green dye with little or no evidence of cell death (Figure 4C).

### GBS interactions with fetal astrocytes

We extended our study to test the hypothesis that following GBS penetration of the *pia mater* meningeal barrier, bacteria interact with astrocyte cells that form the compacted *glia limitans superficialis*. In these experiments, we used a human fetal astrocyte cell line SVGmm [33] and an adult astrocytoma cell line (CCF-STGG1) as *in vitro* models to mimic the *glia limitans*. These cell lines were infected with the normo-haemolytic A909 and hyper-haemolytic NCTC 10/84 strains and bacterial adherence and invasion, cell death and the role of the β-h/c toxin were investigated.

**Figure 5:**
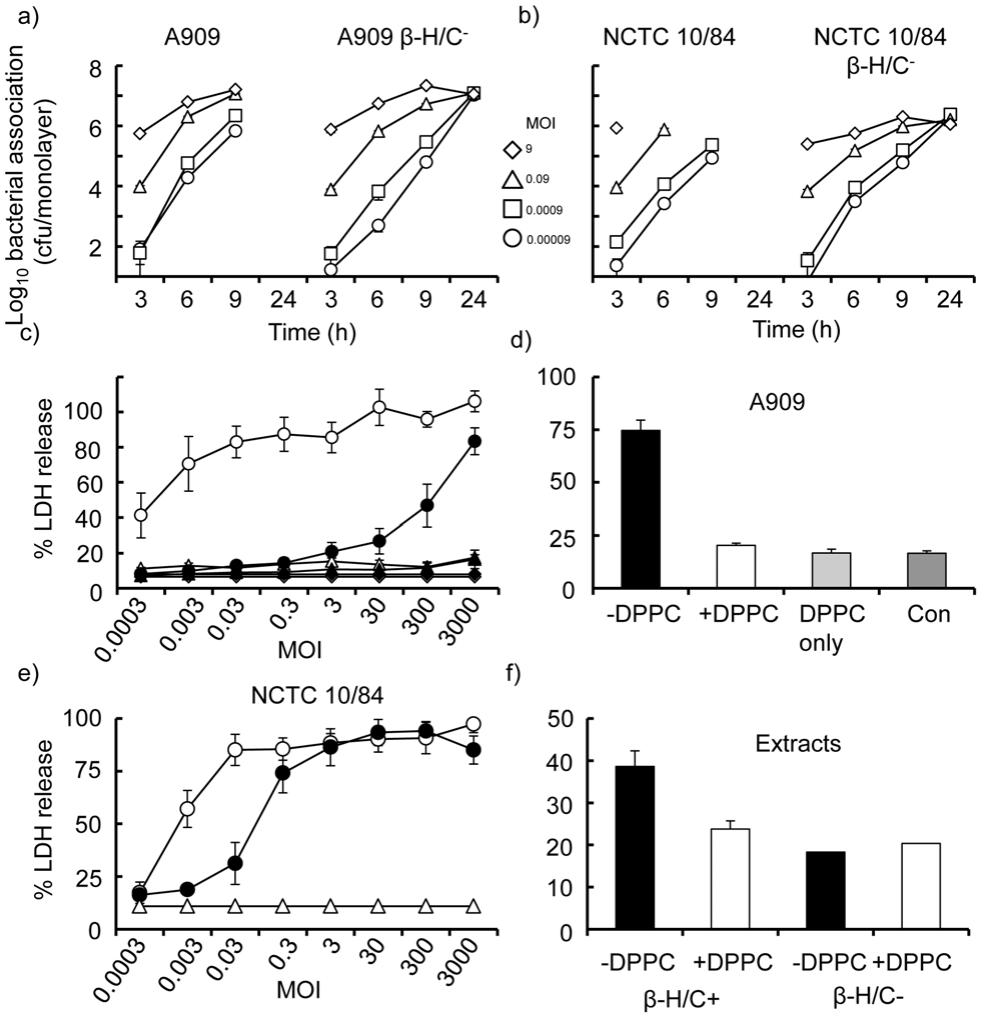
GBS interactions with human fetal astrocytes. a&b) Association of wild-type A909 and NCTC10/84 (β-h/c^+^) strains and their isogenic Δ*cylE* (β-h/c^−^) mutants. SVGmm fetal astrocyte cell monolayers were infected with various initial MOI of GBS strains and association measured over time. Data are from representative experiments (n = 3 for each bacterium) and the symbols show the mean cfu values and the error bars the standard deviations of triplicate wells. c) Astrocyte cell death measured by LDH release. Astrocytes were infected with various initial MOI of wild-type A909 and NCTC10/84 (β-h/c^+^) strains and their isogenic Δ*cylE* (β-h/c^−^) mutants. LDH release was measured after 9 h and the symbols represent the mean levels of LDH release and the error bars the SEM from 3 independent experiments. Open circles denote NCTC10/84 (β-h/c^+^) and open triangles NCTC10/84 Δ*cylE* (β-h/c^−^); closed circles denote A909 (β-h/c^+^) and closed triangles A909 Δ*cylE* (β-h/c^−^); closed diamonds denote background release of LDH. d) Effect of DPPC on astrocyte cell death induced by normo-haemolytic GBS infection. Astrocyte cell monolayers were infected with wild-type strains A909 (initial MOI 3000) in the presence (3 mg/ml) or absence of DPPC, and LDH release measured after 9 h. Control (Con) is spontaneous LDH release from cells. The columns represent mean levels of LDH release and the error bars the SEM from 3 independent experiments. e) Effect of DPPC on astrocyte cell death induced by hyper-haemolytic GBS infection. Cell monolayers were infected with different initial MOI of NCTC10/84 (0.0003–3000) for 9 h in the presence (3 mg/ml) or absence of DPPC. Data are from a representative experiment (n = 3) and the symbols show the mean levels of LDH release and the error bars the standard deviation of triplicate wells. Open and closed circles denote GBS without and with DPPC respectively; open triangles denote background release of LDH. f) Effect of DPPC on β-h/c toxin induced astrocyte cell death. Fetal astrocyte cell monolayers were treated for 9 h with β-h/c^+^ and β-h/c^−^ extract (100 µl volume per well and prepared as described in Materials and Methods) in the presence or absence of DPPC (3 mg/ml). LDH release was measured after 9 h. The columns represent mean levels of LDH release and the error bars the SEM from experiments carried out with 3 independent preparations of toxin extracts.

Both wild-type strains and their Δ*cylE* variants associated with fetal astrocyte cells; in general the dynamics of association for A909 and the β-h/c^−^ mutant were similar for all initial MOI tested up to 9 h, but no monolayers were intact by 24 h with the toxin-expressing strain, whereas they were intact after infection with the mutant (Figure 5a). By contrast, infection with the highest initial MOI of the hyper-haemolytic NCTC 10/84 strain showed higher cytotoxicity than A909 by 3–6 h; the mutant strain, as expected, was not cytotoxic and adherent bacteria were recovered at 24 h (Figure 5b). GBS invasion of SVGmm cells was also an infrequent event. After infection with an initial MOI of 30 for 9 h, the mean invasion rate for A909 (0.3% ±SEM 0.1, n = 3 experiments) was statistically similar (p>0.05) to that observed for the β-h/c^−^ mutant strain (0.49±SEM 0.2, n = 3 experiments). Moreover, these low levels of invasion were similar to those observed with meningioma cells (Table 2). By contrast, invasion could not be estimated at 9 h after infection with an initial MOI of 30 of strain NCTC 10/84, due to cytotoxicity at this time point (Figure 5b), so measurements were made at 6 h. The mean invasion rate for NCTC 10/84 of 0.38% (±SEM 0.05, n = 7 experiments) was significantly lower (p<0.05) than that calculated for the corresponding β-h/c^−^ mutant strain (1.23% ±SEM 0.3, n = 7 experiments). However, as observed with meningioma cells, addition of DPPC (3 mg/ml) during infection of astrocytes led to an increase in the recovery of internalised hyper-haemolytic NCTC 10/84 bacteria, to ∼0.8% (±SEM 0.1, n = 4 experiments).

GBS infection induced high levels of LDH enzyme release by SVGmm astrocyte cells. Infection with an initial MOI of ∼300 of strain A909 was required to induce ≥50% LDH release, which increased to ∼80% with the higher initial MOI of 3000 tested (Figure 5c). The hyper-haemolytic strain was significantly more cytotoxic, as infection with an initial MOI of 0.0003 induced ∼40% LDH release by 9 h and MOI ≥0.003 induced 70–100% LDH release (Figure 5c). As expected, no LDH release was induced by infection with any MOI of the corresponding β-h/c^−^ mutants. We next investigated whether DPPC could inhibit the cytotoxic effect following infection with the parent strains. DPPC (3 mg/ml) significantly inhibited by ∼70% (p<0.05) the release of LDH from SVGmm monolayers infected with strain A909 (initial MOI of 3000) (Figure 5d). However, DPPC was unable to inhibit LDH release (p>0.05) from monolayers initially infected with MOI of 0.3–3000 of the hyper-haemolytic strain, but did inhibit enzyme release by 65–80% after infection with MOI of 0.003–0.03 (Figure 5e). Moreover, DPPC reduced LDH release by ∼50% from cells treated with β-h/c toxin extract (Figure 5f). Similar results were obtained with the adult astrocytoma cell line (data not shown).

## Discussion

GBS are a major cause of neonatal meningitis with high mortality and morbidity rates and in the current study, we used an *in vitro* human meningioma cell culture model of meningitis to investigate the consequences of GBS interactions. Wild type GBS strains demonstrated similar adherence dynamics with meningioma cell monolayers, which were not influenced by either the capsular serotype expressed or by capsule expression. We also demonstrated that GBS strains adhered to astrocytes. In particular, adherence to both cell types was not influenced by β-h/c toxin expression. By contrast, previous studies have shown that capsule expression and β-h/c toxin could influence GBS-host cell interactions *in vitro*, but this depended on the cell type and moreover, the methods to measure adherence were not comparable [14], [34]–[37]. The adhesins used by GBS for adherence to meningioma cells and astrocytes remain to be identified, but any one or more GBS components such as PilA [38], [39], lipoteichoic acid [40], C5a peptidase [41], FbsA [42], alpha-C protein and the HvgA protein [19], which mediate adherence to epithelial and endothelial cells, merit investigation.

A general property of GBS bacteria *in vivo* and *in vitro* is their ability to penetrate human cellular barriers, particularly those composed of chorion cells [43], lung epithelial cells [37], [44] and BMECs [13]. However, direct invasion of meningioma cells by GBS was a slow and infrequent event, with very low levels of intracellular bacteria detected by 9 h. Nevertheless, this very low level of invasion was influenced by GBS capsule expression, which has been similarly observed during invasion of cells of the respiratory tract and endothelium [13], [34], [45]. Direct invasion of astrocytes by GBS strains was also a rare event; moreover, for both cell types invasion was not influenced by β-h/c toxin expression. By contrast, toxin expression has been shown to play a role during GBS invasion of lung epithelial cell cultures *in vitro* [37] and of endothelial cells in a murine model of meningitis [14]. In particular, GBS β-h/c^+^ bacteria penetrated endothelial cells and established meningitis in a murine model more frequently than the corresponding β-h/c^−^ mutant [14].

Our study also showed that GBS growth, β-h/c toxin expression and interactions with human cells resulted in host cell death. Notably, astrocytes were more sensitive to the cytotoxic effects of infection than meningioma cells and the effect was more pronounced with the hyper-haemolytic isolate NCTC 10/84. Indeed, there appeared to be a spectrum of toxin production by the different GBS clinical isolates and although the hyper-haemolytic strain is likely atypical, it did confirm that excess β-h/c toxin production was deleterious. This isolate and an initial MOI of 3000 are likely to reflect the extremes of GBS infection. Interestingly, with this high MOI (3000), meningioma cell LDH levels were reduced by all GBS strains, except for A909. This difference cannot be accounted for by variation in bacterial growth rates, since these were similar for all wild-type and mutant GBS strains. Given that GBS did not degrade LDH enzyme, the absence of measurable LDH after infection with an initial MOI of 3000 could be due to degradation by rapidly released meningioma cell autolytic enzymes. It is also generally believed that the β-h/c toxin is attached to the surface of the bacterial cell [46]: however, it is not known whether the levels of attached toxin vary between strains, possibly as a consequence of differences in transport to the cell membrane and/or toxin stability. Thus, host cells remain viable after A909 infection, perhaps due to such intrinsic variations. Molecular characterization of the β-h/c toxin, including transport to the cell membrane, stability and relationship to pigmentation, still proves elusive and requires further study.

The β-h/c toxin plays a role in meningioma and astrocyte cell death, which has also been observed for GBS-infected epithelial cells and BMECs [13], [32], [37]. Evidence for this role was provided by the findings that wild-type bacteria and β-h/c^+^ extracts induced cell death, whereas challenge with Δ*cylE* (β-h/c^−^) mutant strains and β-h/c^−^ extracts did not. DPPC also significantly reduced meningioma cell injury induced by wild-type bacteria and β-h/c^+^ extracts. Although protection was also observed for astrocytes infected with normo-haemolytic GBS, DPPC was not wholly effective at inhibiting cytotoxicity caused by the hyper-haemolytic GBS strain or β-h/c^+^ extracts. These observations suggest that the increased toxicity of GBS towards astrocytes could involve GBS virulence factors in addition to β-h/c toxin. It is also possible that astrocytes express receptors that specifically recognise these other GBS virulence factors, but which are absent on meningioma cells.

Previous studies have shown protective effects of DPPC against β-h/c mediated injury in epithelial cells [32], murine macrophages [47] and cardiomyocytes [48]. It has been suggested that DPPC might preserve the host cell membrane by providing phospholipid replacement during pore formation and/or by direct neutralisation by binding to toxin itself [37]. Or, like pneumococci, GBS possibly trigger cell death by blocking the biosynthesis of host phosphatidylcholine [49] and this is reversed by the addition of exogenous lipid. Whether any of these mechanisms are functional during meningeal cell protection remains to be confirmed and to our knowledge there are no data on the potential therapeutic benefit of surfactant phospholipids in GBS meningitis. However, surfactant treatment that maintains monolayer integrity might be of benefit during GBS meningitis as a means to preserve an intact *pia mater* barrier to bacterial penetration to sub-pial tissue.

A significant finding from our study was that GBS infection did not induce a cytokine response from either meningioma cells or astrocytes (Tables S1, S2, S3). It is possible that GBS-induced rapid host cell death prevented *de novo* cytokine protein synthesis: however, cytokines were also not induced following infection with β-h/c^−^ mutant bacteria, weakly-haemolytic bacteria or heat-killed bacteria, all of which allowed prolonged host cell viability (Tables S1, S2, S3). Possible explanations include GBS inhibition of cytokine production by these cells, which has been observed for meningioma cells following *S. pneumoniae* infection *in vitro* [31] and/or proteolytic degradation of cytokines, a property that has been shown for the GBS serine protease CspA [50].

The combined effects of GBS on meningeal cells and astrocytes *in vitro* contribute to our understanding of the pathophysiology of GBS neonatal meningitis. Our study shows that GBS rapidly grow and colonise meningeal cells and *in vivo* these events are most likely unchecked by an immature intracranial immune system [51]. We also show that by failing to secrete cytokines in response to infection, the meninges are unlikely to be involved in innate inflammatory responses to GBS. Thus, inflammatory mediators observed in the CSF of neonates with GBS meningitis are probably produced by damaged endothelial cells, by the small population of perivascular and meningeal resident macrophages and by infiltrating immune cells [52], [53]. Our study also demonstrates that meningeal cells provide a significant barrier to intracellular penetration by GBS bacteria, but this barrier is circumvented by GBS β-haemolysin/cytolysin-induced cell death. Penetration of the *pia* mater would allow GBS to gain access to the sub-pial space and then interact with the compacted astrocytes of the underlying *glia limitans superficialis*. Our study shows that GBS adhere to fetal astrocytes and rapidly kill these cells without significant intracellular invasion.

Thus, the events occurring in the meninges and *glia limitans* could contribute to the significant damage to underlying brain tissue that is observed in humans and in animal models of GBS meningitis [54], [55]. This damage correlates with mortality and the high levels of permanent neurological sequelae observed in survivors [6] GBS infection can cause encephalomalacia (softening of brain tissue) and vasculopathy and post-mortem examination of a neonate with GBS meningitis showed a loss of brain structural integrity with a thin purulent exudate on the surface of the brain and ventricles [56], [57]. Deeper penetration through necrotic tissue is also likely to induce Toll-like receptor 2-dependent microglial cell activation and subsequent apoptosis [58] and a murine model of GBS meningitis has shown brain tissue destruction and neutrophil infiltration [54]. In a rat model, GBS infection was reported to induce a caspase-3-independent form of cell death, characterised by neuronal loss in the dentate gyrus of the hippocampus [59]. In addition, a role has been shown for the β-h/c toxin in inducing cell injury in the cortex and hippocampus of rats [55].

In summary, we have used *in vitro* cell culture based models of meningitis to investigate the interactions of GBS with meningeal cells and astrocytes. Understanding the mechanisms involved will help to identify potential therapies for improving patient survival and for reducing the severity of neurological sequelae.

## Materials and Methods

### Bacterial strains and growth conditions

In this study, 10 strains of Group B Streptococcus (GBS) of different capsular types were used (Table 1). Presence of the Group B capsular polysaccharide was confirmed on all GBS strains by serology using the Streptococcal grouping kit (Oxoid, Basingstoke, UK). Strains A909, H36B, 18RS21, 2603V/R and COH-1 were obtained from the American Type Culture Collection (ATCC) (LGC Promochem, Teddington, UK). Additional wild type GBS strains and their isogenic mutants that are deficient in β-haemolysin/cytolysin (β-h/c) and capsule production have been described elsewhere (Table 1). *Neisseria meningitidis* MC58 serogroup B strain was isolated in Stroud, Gloucestershire in the mid-1980s from an outbreak of meningococcal infections [60] and *Escherichia coli* IH3080 serogroup O18:K1 is a clinical isolate from a neonate presenting with meningitis [61] and was obtained from the National Institute of Public Health, Helsinki, Finland. GBS strains, *N. meningitidis* and *E. coli* were grown directly from liquid nitrogen stocks onto Brain Heart Infusion (BHI) agar, GC agar, and Luria-Bertani (LB) agar respectively. Organisms were grown overnight in a humidified incubator with a 5% (v/v) CO_2_ atmosphere at 37°C.

### Preparation of β-haemolysin/cytolysin (β-h/c) extract from GBS

The β-h/c toxin was prepared following the method as originally described by Marchlewicz and Duncan [62]. GBS strains were grown overnight on BHI agar plates and a few colonies were emulsified in 1.2 ml of Todd Hewitt Broth (THB) (Oxoid, UK), which was grown overnight as a static culture in a humidified incubator with a 5% (v/v) CO_2_ atmosphere at 37°C. This starter culture was then inoculated into a total volume of 25 ml of fresh THB and this culture was incubated until an O.D of 0.8 at λ600 nm was reached. The bacteria were centrifuged (3000 g, 5 min), the pellet was washed twice in warm PBS and then suspended in 3 ml of warm PBS solution containing 0.2% (w/v) glucose and 1% (w/v) starch, followed by incubation for 90 min at 37°C in an orbital shaking incubator (Gallenkamp, UK, 100 rpm). The suspension was then centrifuged (3,000*g*, 5 min) and the supernatant was sterilised using 0.22 µm PES (polyethersulfone) filters (Millipore, USA) and was kept on ice throughout the process. The filtered extract was divided into several working aliquots, which were stored at −80°C and used within 2 days.

The haemolytic activity of the β-h/c extracts was determined using sheep red cells [62]. Using 50 µl of crude extract, a two-fold serial dilution of the extract was prepared using PBS in a U-shaped 96-well measuring plate (Sterilin, UK). Then, 50 µl of 1% (v/v) suspension of sheep red cells (Oxoid, UK) was added to all wells, followed by 100 µl of PBS. Negative and positive controls were included by adding 150 µl of PBS (negative control) and 150 µl of distilled water (positive control) to 50 µl of red cell suspension. The final volume in all wells was 200 µl. The plate was incubated at 37°C for 30 min, followed by storage at 4°C for 2 h. Haemoglobin (Hb) release from the red cells was then measured by transferring the supernatant from each well to a measuring plate, which was read at λ420 nm using an iMark plate absorbance reader (Bio-Rad, USA). Hb release was expressed as a percentage of total release of the positive control using the following formula: Hb release of sample (%) = (A_420_ of sample/A_420_ of positive control)×100. The end point (titre) of the crude extract was defined as the highest dilution of extract that induced 50% release of Hb from the red cell suspension. The number of haemolytic units (HU) per ml of extract was determined in order to use a known concentration of HU in subsequent experiments. A haemolytic unit of 1.0 was defined as the volume (highest dilution) of crude extract that induced 50% Hb release from 1 ml of 1% (v/v) red cell suspension [62].

### Culture of human meningioma cells and astrocytes

Human meningioma cells were obtained from surgically removed tumours as described previously [28]. For this study, we used meningioma cell lines (n = 2) that were generated in a previous study and shown to express the characteristic markers of desmosomal desmoplakin, epithelial membrane antigen, vimentin and cytokeratin [63]. The cells were grown in Dulbecco's modified Eagles medium (DMEM) with Glutamax-1 and sodium pyruvate (Lonza, Slough, UK) supplemented with 10% (v/v) heat-inactivated, decomplemented foetal calf serum (Lonza) (dFCS). Cells were seeded into T75 cm^2^ flasks (greiner bio-one, Frickenhausen, Germany) pre-coated with collagen (type I from rat tail, 5 µg/cm^2^; Becton Dickinson, UK). Cells were maintained in a 5% (v/v) CO_2_ atmosphere at 37°C and were grown to confluence and culture passages from 4–10 were used in the experiments. The human fetal astrocyte cell line SVGmm was grown in Eagle's Minimal Essential Medium (EMEM, Lonza) supplemented with 10% (v/v) dFCS [33]. The adult astrocytoma cell line CCF-STTG1 (CRL-1718; LGC Promochem, UK) was grown in Rosewell Park Memorial Institute medium containing 10% (v/v) dFCS.

### Reagents

A10x solution of the phospholipid surfactant dipalmitoylphosphatidylcholine (DPPC) was prepared fresh by dissolving 30 mg of DPPC (Sigma-Aldrich, Dorset, UK) in 1 ml of DMEM or EMEM medium containing 1% (v/v) dFCS, with sonication at 30 micrometers for 2×30 sec bursts (MSE Soniprep).

### Challenge of human meningioma cells and astrocytes

#### i) Measurement of total bacterial association

Human meningioma cells and astrocytes were grown to confluence in 24-well cell culture plates. The average meningioma cell count in a 24-well plate was 3.3×10^4^/well (±1×10^4^/well, n = 7) and for SVGmm astrocytes, 1.1×10^5^/well (±0.25×10^5^/well, n = 6). Before bacterial challenge, the cell monolayers were maintained overnight in medium containing 1% (v/v) dFCS and then they were washed gently twice with warm PBS (pH 7.4). GBS suspensions were prepared in DMEM or EMEM containing 1% (v/v) dFCS from fresh overnight cultures of bacteria grown on BHI agar plates. Cell monolayers were challenged in triplicate with bacteria, with 1 ml volume per well. The initial multiplicity of infection (MOI, number of bacteria/average number of cells per monolayer) for meningioma cells was 0.0003 (10^1^ cfu/monolayer) 0.003 (10^2^), 0.3 (10^4^), 30 (10^6^), 300 (10^7^) to 3000 (10^8^). For astrocytes, the initial MOI was 0.00009 (10^1^), 0.0009 (10^2^), 0.09 (10^4^), 9 (10^6^) and 900 (10^8^). Monolayers were incubated at time intervals up to 24 h.

In order to measure total bacterial association with cells, monolayers were washed gently 4 times with warm PBS, and then visually checked for integrity under an inverted light microscope. A sterile lysis solution of PBS containing 1% (w/v) saponin (Sigma-Aldrich) was added to the wells (250 µl/well) and incubated for 15 min at 37°C. Bacteria were quantified as viable counts by inoculating the lysate at appropriate dilutions onto BHI agar plates in triplicate and incubating overnight at 37°C. Meningioma cell monolayers were also challenged with *N. meningitidis* MC58 (MOI of 0.3), and viable counts were done on GC agar plates. A two-sample student t-Test was used to compare the mean levels of total bacterial association between GBS strains, with a value of P<0.05 determining significance.

#### ii) Measurement of bacterial invasion

Human meningioma cells and astrocyte monolayers, established in 24-well cell culture plates, were challenged in triplicate with bacterial suspensions of initial MOI 0.3 and 0.09 (10^4^ cfu/monolayer) respectively, and incubated for up to 9 h. The measurement of bacterial invasion was carried out using the gentamicin-cytochalasin D (CD) assay as previously described [28]. Gentamicin effectively killed concentrations of GBS bacteria at growth levels expected in the culture medium after 9 h (data not shown) and has been reported to show limited penetration of human cells [64], [65]. The involvement of host cell actin polymerisation was investigated by adding CD to the monolayers (to a final concentration of 1 µg/ml), 30 minutes before GBS infection. In addition, *E. coli* IH3080 was included in the invasion experiments as a positive control strain, since it has been previously shown to invade human meningioma cells [31]. The percentage of invading bacteria (invasion rate) was calculated using the formula: Invasion rate (%) = (internalised bacteria/associated bacteria)×100. A two-sample student t-Test was used to compare the mean levels of invasion in the presence and absence of treatments, with a value of P<0.05 determining significance.

### Electron microscopy

#### i) Scanning Electron Microscopy (SEM)

Human meningioma cells were grown to confluence on collagen pre-coated transwells (ThinCerts™, transparent, pore size 0.4 µm; greiner bio-one, Germany) and were challenged for up to 24 h with an initial MOI of 0.3 (10^4^ cfu/ml) bacteria. Uninfected cells were included as negative controls. At different time points, the cells were washed 4 times with warm PBS to remove unbound bacteria and were then processed for SEM. Transwells were fixed for a minimum of 1 h in 3% (v/v) glutaraldehyde and 4% (v/v) formaldehyde in 0.1 M PIPES (1,4-Piperazinediethanesulfonic acid) buffer, pH 7.2. Then, a gradual concentration of ethanol was used to dehydrate the samples and the transwell membrances were cut during dehydration. The cut membranes were critical-point dried and mounted onto metal stubs and the coated specimens were then viewed using the Quanta 200 scanning electron microscope (FEI, USA).

#### ii) Transmission Electron Microscopy (TEM)

Human meningioma cells were grown in T75 cm^2^ tissue culture flasks (greiner bio-one, Germany) to confluence (1×10^6^ cells/flask ±2×10^5^, n = 3).Monolayers were infected with 15 ml of bacterial suspensions containing 2×10^4^ cfu/ml, which was equivalent to the initial MOI (0.3) used in invasion experiments. At a given time point, the monolayers were washed 4 times with warm PBS and were gently scraped off from the flask in the presence of PBS using a cell scraper. For TEM processing, the cell harvest was fixed using a solution containing 3% (v/v) glutaraldehyde+4% (v/v) formaldehyde in 0.1 M PIPES buffer, pH 7.2 for a minimum of 1 hr. The cells were mixed with 5% (w/v) sodium alginate and ejected into a 0.1M calcium chloride solution to form specimen balls. The specimen balls were treated for 1 h with a post fixative solution of 1% (w/v) osmium tetroxide in 0.1M PIPES buffer, pH 7.2. Samples were briefly rinsed twice in water, and then a 2% (w/v) uranyl acetate solution was used for staining for 30 min. A series of gradual concentrations of ethanol was used to dehydrate the samples. Samples were incubated overnight in spur resin at a 50∶50 ratio with acetonitrile. The samples were finally placed in fresh spur resin and polymerised in an electric oven at 60°C for 20–24 h. In preparation for microscopy, sample blocks were cut into thin sections using a Leica Reichert Ultracut E microtome (Leica, Germany) using glass knifes and several sections per sample were fixed onto copper grids. Grids were stained using lead nitrate for 5 min and then examined using the H7000 transmission electron microscope (Hitachi, Japan) at 75 Kv and under different magnifications ranging from ×3000 to ×20,000.

### Host cell cytotoxicity assays

#### i) Release of lactate dehydrogenase (LDH)

Human meningioma cells and astrocytes were grown to confluence in 96-well tissue culture plates. The average meningioma cell count was 6.8×10^3^cells/well (±8×10^2^, n = 6) and for astrocytes, 3.7×10^4^cells/well (±9.6×10^3^, n = 6). Bacterial inocula were prepared in DMEM or EMEM containing 1% (v/v) dFCS at concentrations that yielded initial MOIs from 0.0003 to 3000. The monolayers were washed twice with warm PBS and then 200 µl/well of each bacterial inoculum was added to triplicate wells. Plates were incubated in an atmosphere of 5% (v/v) CO_2_ at 37°C and LDH release was measured at intervals up to 9 h using the CytoTox 96® Non-Radioactive Cytotoxicity assay kit (Promega, UK) according to the manufacturer's protocol. For controls, uninfected monolayers were included for measurements of Spontaneous LDH release and Maximum LDH release induced by the addition of proprietary lysis reagent. The absorbance was read at λ492 nm (iMark Absorbance Reader, Bio-Rad, USA). Cytotoxicity levels were calculated by dividing the average LDH release value of test by the average Maximum LDH release value (from lysed, uninfected cells) using the following formula: LDH release of test (%) = (LDH release of test/Maximum LDH release)×100.

In order to determine the effect of DPPC on the release of LDH, bacterial suspensions that induced the highest LDH release were used to infect cell cultures in the presence of DPPC (3 mg/ml) in a final volume of 200 µl/well. In order to determine the effect of the β-h/c^+^ extract on the release of LDH, 100 µl of extract containing 50–100HU and 100 µl of medium was added to the monolayers. DPPC solution was also added to appropriate wells at a final concentration of 3 mg/ml, instead of medium. For additional controls, infected or extract-treated wells with no DPPC were included. Extracts from mutant strains were also tested in the experiments and were added to monolayers in equivalent volumes to the β-h/c^+^ extracts.

The effects of the DPPC on bacterial interactions were investigated by adding DPPC solution (3 mg/ml) to the meningioma and astrocyte cells at the time of infection. Total bacterial association and internalised bacteria were determined up to 9 h as described above and untreated monolayers were included at each time point for comparison. DPPC did not affect the viability of either cell cultures or the growth of GBS strains (p>0.05) or the ability of bacteria to associate with the cells (p>0.05; Figure S4A, S4B). In order to confirm that DPPC itself was not interfering with the enzyme-substrate reaction, control monolayers were treated with DPPC (3 mg/ml) alone and then lysed after 9 h and LDH release measured. In these controls, LDH release was similar to the Maximum LDH release values, demonstrating that incubation with DPPC did not interfere with the assay (data not shown). In addition, LDH enzyme was not degraded by GBS bacteria (data not shown). A two-sample Student T-test was used to compare mean LDH release induced by bacteria or β-h/c extract, with and without DPPC or against mutants lacking β-h/c; a value of P<0.05 was considered significant.

#### ii) Meningioma cell viability determined using the LIVE/DEAD® assay

Meningioma cells were grown to confluence in collagen pre-coated transwells. Suspensions of GBS strains were prepared in medium containing 1% (v/v) dFCS and then added to the monolayers at initial MOIs ranging from 0.03–3000 bacteria/cell. Uninfected cells were included in each experiment as negative controls. In order to visually demonstrate the effect of DPPC on the viability of meningeal cells infected with live GBS strains, confluent cells were infected with bacteria in the presence and absence of DPPC (3 mg/ml). In addition, the effect of β-h/c extracts on cell death was visualised after treatment with 250HU of toxin in the presence and absence of DPPC (3 mg/ml).

The plates were placed in a humidified incubator with a 5% (v/v) CO_2_ atmosphere at 37°C and at intervals up to 9 h, the monolayers were washed twice in warm PBS and then stained using the LIVE/DEAD® Viability/Cytotoxicity assay (Molecular Probes, UK) according to the manufacturer's instructions. The fluorescent staining was viewed under a Leica SP-2 laser-scanning confocal microscope system (Leica, Germany). Images were obtained by simultaneous two-channel scanning at 488 and 568 nm to excite the FITC and PI signals, which are similar to the calcein AM (green dye) and ethidium homodimer (red dye) markers used in the assay. Maximum projection images were subsequently prepared from 25 optical sections obtained at a magnification of ×40 under oil immersion.

### Measurement of cytokine production

The measurement of pro-inflammatory cytokine IL-6, chemokines IL-8, MCP-1 and RANTES was carried out by immunosorbent assay (sandwich technique) as described previously [66]. The innate response of the monolayers, within the same experiments, was demonstrated by induction of cytokine secretion induced by infection with *N. meningitidis* strain MC58, as described previously [67].

## Supporting Information

Figure S1 Measuring the levels of GBS adherence to plastic and relative growth rates. A) In order to demonstrate that GBS did not adhere significantly to plastic surfaces, control experiments were done in which GBS strains A909 (Ia), 18RS21 (II), NEM316 (III) and 2603V/R (V) (10^1^,10^2^, 10^4^, 10^6^ cfu/well) were grown in DMEM medium containing 1% (v/v) dFCS in 24 well plates without human cells. At intervals up to 24 h, samples were taken for bacterial growth and adherence to plastic was quantified after washing with PBS and saponin lysis as per the standard adhesion assay. The symbols describe the mean cfu levels and the error bars the standard deviation of triplicate wells. For the other strains, measurements are only shown at 9 h. The level of adherence of GBS is shown on each figure and is consistently <0.5% of GBS bacterial growth in the wells, at any time point sampled during growth. B) Growth curves of GBS wild-type and mutant strains. GBS strains were inoculated (10^2^ cfu/ml) into DMEM medium containing 1% (v/v) dFCS and bacterial growth determined over time by viable counting. All growth rates were statistically similar between wild-type strains and between wild-type strains and mutant strains (p>0.05).
(TIF)

Figure S2 Electron microscopy of human meningioma cells infected with GBS bacteria. (A) Scanning electron microscopy of cells infected with an initial MOI of 0.3 (10^4^ cfu/monolayer) of wild type GBS (serotype III strain NEM316) over time (3–24 h). Low and high magnification images are shown for 9 h. Similar images were obtained for other GBS serotypes (not shown). (B) Transmission electron microscopy of cells infected with GBS bacteria. Cell monolayers were infected with an intial MOI of 0.3 (10^4^ cfu/ml) of wild-type strain A909 and processed for TEM after 9 h. (1) Control uninfected meningioma cell. (2) GBS bacterium contained within a cell process. (3) Intracellular GBS bacterium within a vacuole. Images obtained after examining ≥25 grids per sample (×6000 magnification).
(TIF)

Figure S3 Treatment of monolayers with cytochalasin D (CD) reduces the recovery of internalised GBS after gentamicin treatment. Meningioma cell monolayers were initially treated with cytochalasin D (CD) and then infected with GBS strains (initial MOI 0.3) for 9 h. Monolayers with and without CD pre-treatment were washed and gentamicin added to remove extracellular and surface-bound bacteria. Viable counts of internalised bacteria were made following saponin lysis. The columns represent the mean values of recovered bacteria with or without CD treatment and the error bars the SEM from 3–6 independent experiments.
(TIF)

Figure S4 DPPC dose-dependent reduction of LDH release from GBS-infected meningioma cells. A) Various doses of DPPC were added to meningioma cells on challenge (t = 0 h) with GBS strain A909 (initial MOI 3000). LDH release was measured after 9 h. As controls, cells were infected without DPPC and were also maintained without infection in the presence of increasing amounts of DPPC. The columns show the mean level of LDH release and the error bars the SEM from n = 2 experiments. B) DPPC does not affect the growth of GBS or bacterial association with meningioma cells. Monolayers of meningioma cells were infected with strain NCTC10/84 in the presence or absence of DPPC (3 mg/ml) and bacterial growth in the medium and association with human cells was quantified after 9 h. The columns represent mean viable counts of bacteria and the error bars the SEM from at least 3 independent experiments.
(TIF)

Table S1 Infection of meningioma cells with GBS does not induce cytokine secretion. Meningioma cell lines (n = 2) were infected with various MOI of the GBS strains and cytokine secretion measured by ELISA after 24 h. As a control, cells were also infected with *Neisseria meningitidis* strain MC58 and wells were also left with medium alone (uninfected). The data are the mean levels of cytokine secretion (ng/ml) with the standard error of the mean (SEM in parenthesis) from n = 3 independent experiments.
(DOCX)

Table S2 Infection of meningioma cells by heat-killed GBS does not induce cytokine secretion. Meningioma cells were infected with heat-killed GBS strain NEM316 and cytokine production measured after 24 and 48 h. Data are the mean levels of cytokine secretion (ng/ml) with standard deviation (SD) of n = 3 wells and they are similar to data presented for viable bacteria in Table S1 for NEM316. Heat-killed MC58 bacteria show a reduced capacity to induce cytokines compared to viable bacteria (Table S1).
(DOCX)

Table S3 Infection of SVGmm astrocytes with GBS does not induce cytokine secretion. Astrocyte cell lines (n = 3 experiments) were infected with various MOI of the GBS strains A909 and A909Δ*cylE* and cytokine secretion measured by ELISA after 24 h. As a control, cells were also infected with *Neisseria meningitidis* strain MC58 and wells were also left with medium alone (uninfected). The data are the mean levels of cytokine secretion (ng/ml) with the standard deviation (in parenthesis) of triplicate wells from a representative experiment.
(DOCX)
