# Supplementary figures and images for: Group B Streptococcus Interactions with Human Meningeal Cells and Astrocytes In Vitro
Source: PLoS One. 2012 Aug 10;7(8):e42660. doi: 10.1371/journal.pone.0042660 (PMC3416839; doi:10.1371/journal.pone.0042660)

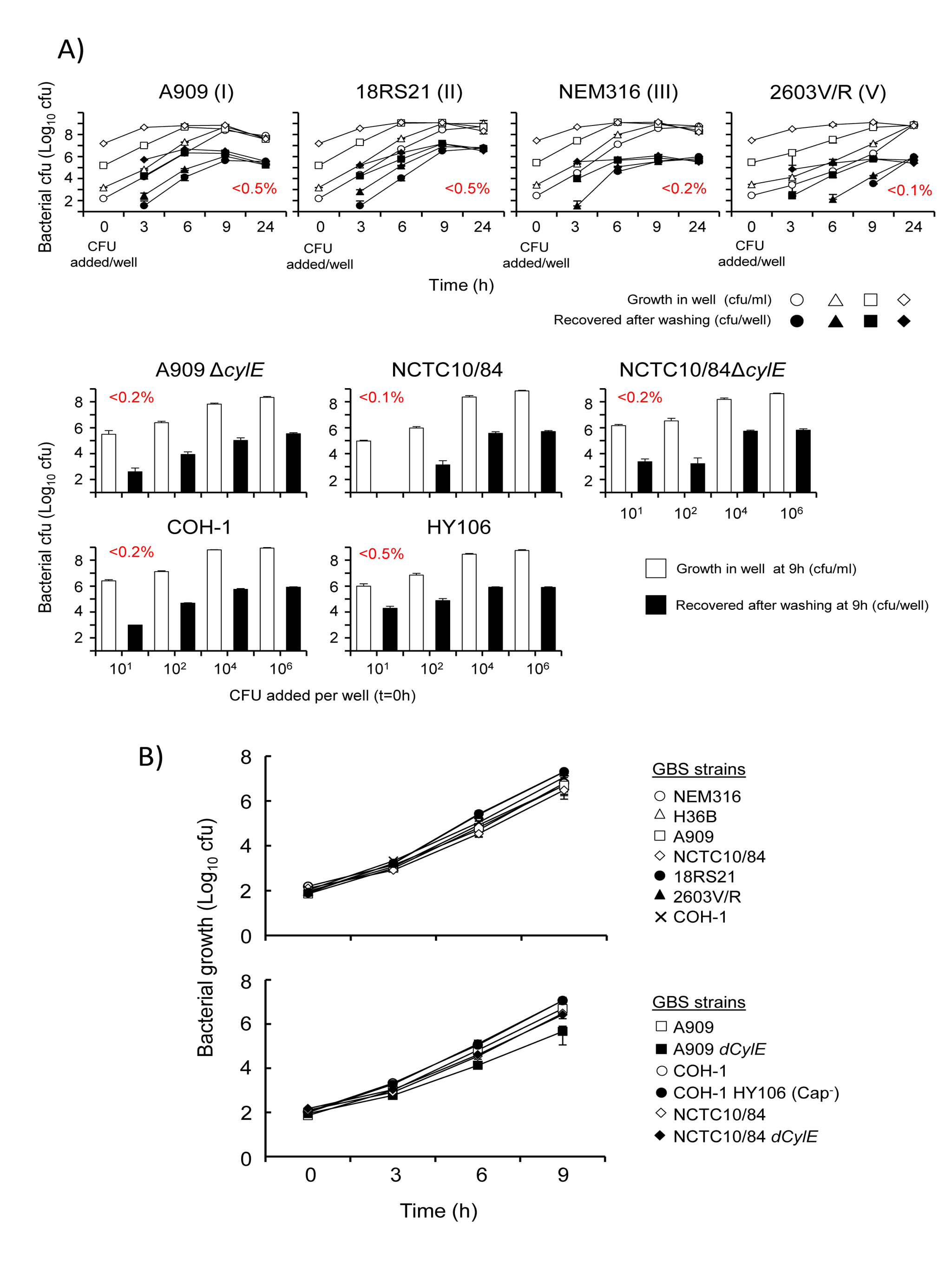

Supplement: Figure S1 — Measuring the levels of GBS adherence to plastic and relative growth rates. A) In order to demonstrate that GBS did not adhere significantly to plastic surfaces, control experiments were done in which GBS strains A909 (Ia), 18RS21 (II), NEM316 (III) and 2603V/R (V) (101,102, 104, 106 cfu/well) were grown in DMEM medium containing 1% (v/v) dFCS in 24 well plates without human cells. At intervals up to 24 h, samples were taken for bacterial growth and adherence to plastic was quantified after washing with PBS and saponin lysis as per the standard adhesion assay. The symbols describe the mean cfu levels and the error bars the standard deviation of triplicate wells. For the other strains, measurements are only shown at 9 h. The level of adherence of GBS is shown on each figure and is consistently <0.5% of GBS bacterial growth in the wells, at any time point sampled during growth. B) Growth curves of GBS wild-type and mutant strains. GBS strains were inoculated (102 cfu/ml) into DMEM medium containing 1% (v/v) dFCS and bacterial growth determined over time by viable counting. All growth rates were statistically similar between wild-type strains and between wild-type strains and mutant strains (p>0.05). (TIF) [file pone.0042660.s001.tif]

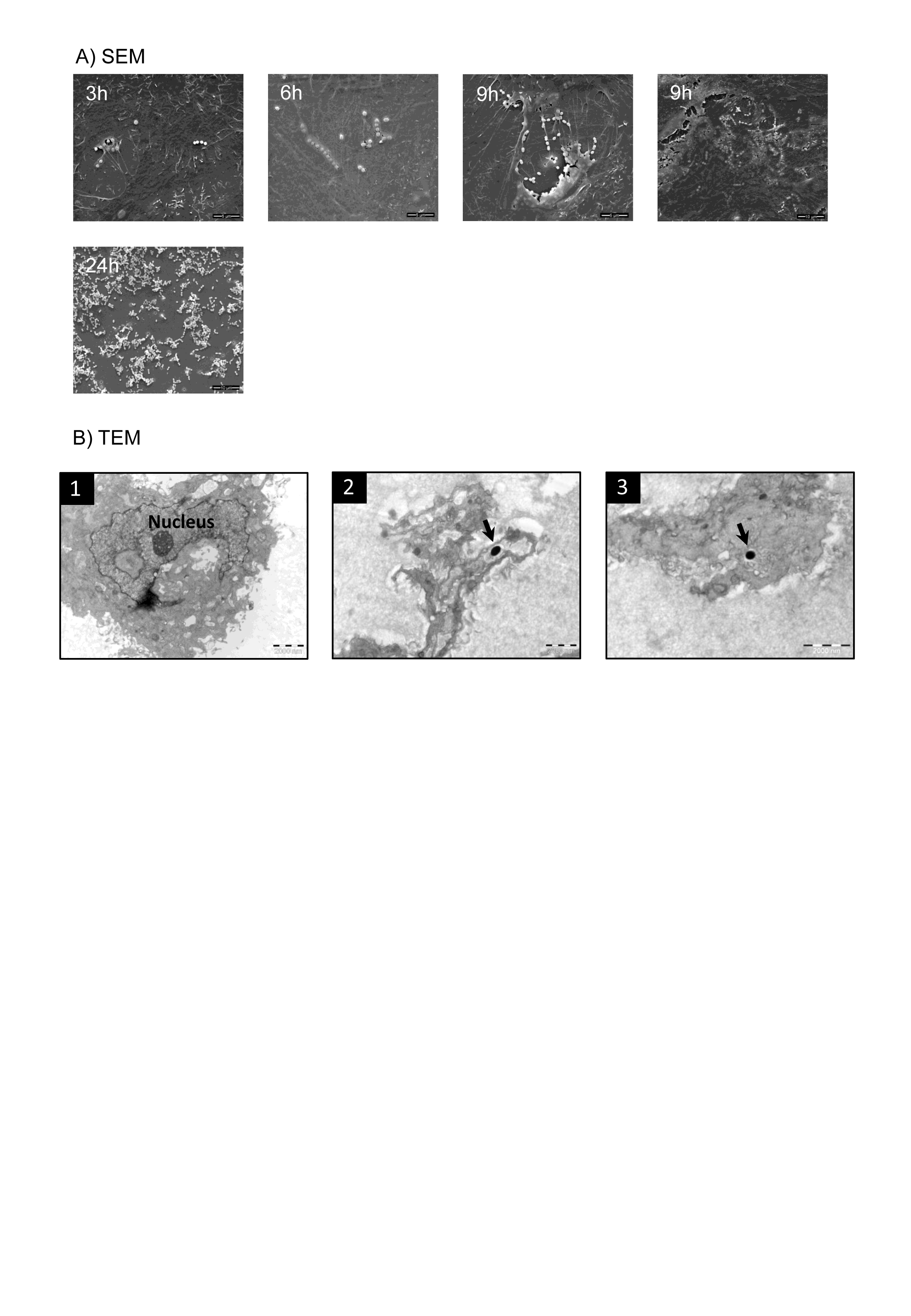

Supplement: Figure S2 — Electron microscopy of human meningioma cells infected with GBS bacteria. (A) Scanning electron microscopy of cells infected with an initial MOI of 0.3 (104 cfu/monolayer) of wild type GBS (serotype III strain NEM316) over time (3–24 h). Low and high magnification images are shown for 9 h. Similar images were obtained for other GBS serotypes (not shown). (B) Transmission electron microscopy of cells infected with GBS bacteria. Cell monolayers were infected with an intial MOI of 0.3 (104 cfu/ml) of wild-type strain A909 and processed for TEM after 9 h. (1) Control uninfected meningioma cell. (2) GBS bacterium contained within a cell process. (3) Intracellular GBS bacterium within a vacuole. Images obtained after examining ≥25 grids per sample (×6000 magnification). (TIF) [file pone.0042660.s002.tif]

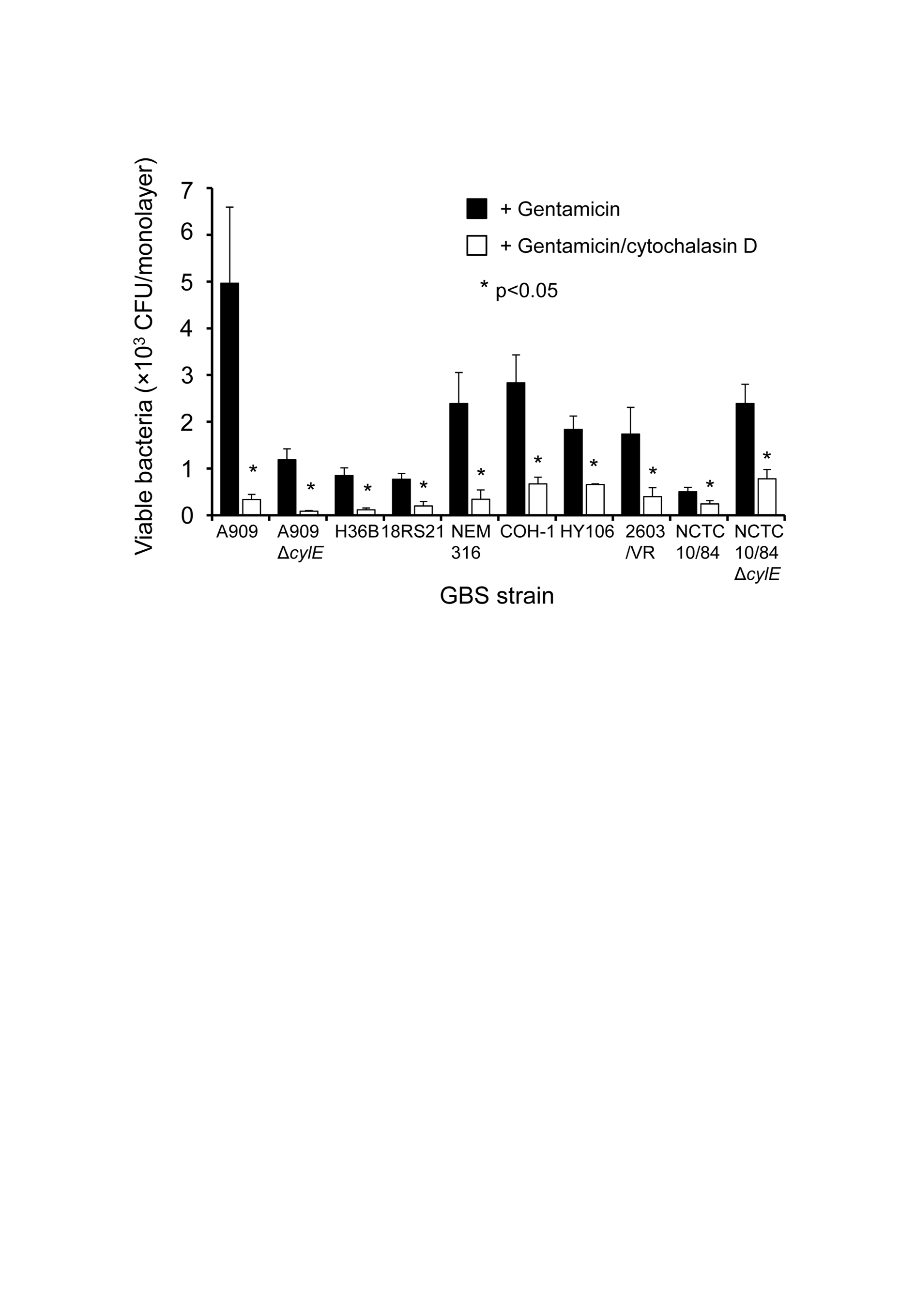

Supplement: Figure S3 — Treatment of monolayers with cytochalasin D (CD) reduces the recovery of internalised GBS after gentamicin treatment. Meningioma cell monolayers were initially treated with cytochalasin D (CD) and then infected with GBS strains (initial MOI 0.3) for 9 h. Monolayers with and without CD pre-treatment were washed and gentamicin added to remove extracellular and surface-bound bacteria. Viable counts of internalised bacteria were made following saponin lysis. The columns represent the mean values of recovered bacteria with or without CD treatment and the error bars the SEM from 3–6 independent experiments. (TIF) [file pone.0042660.s003.tif]

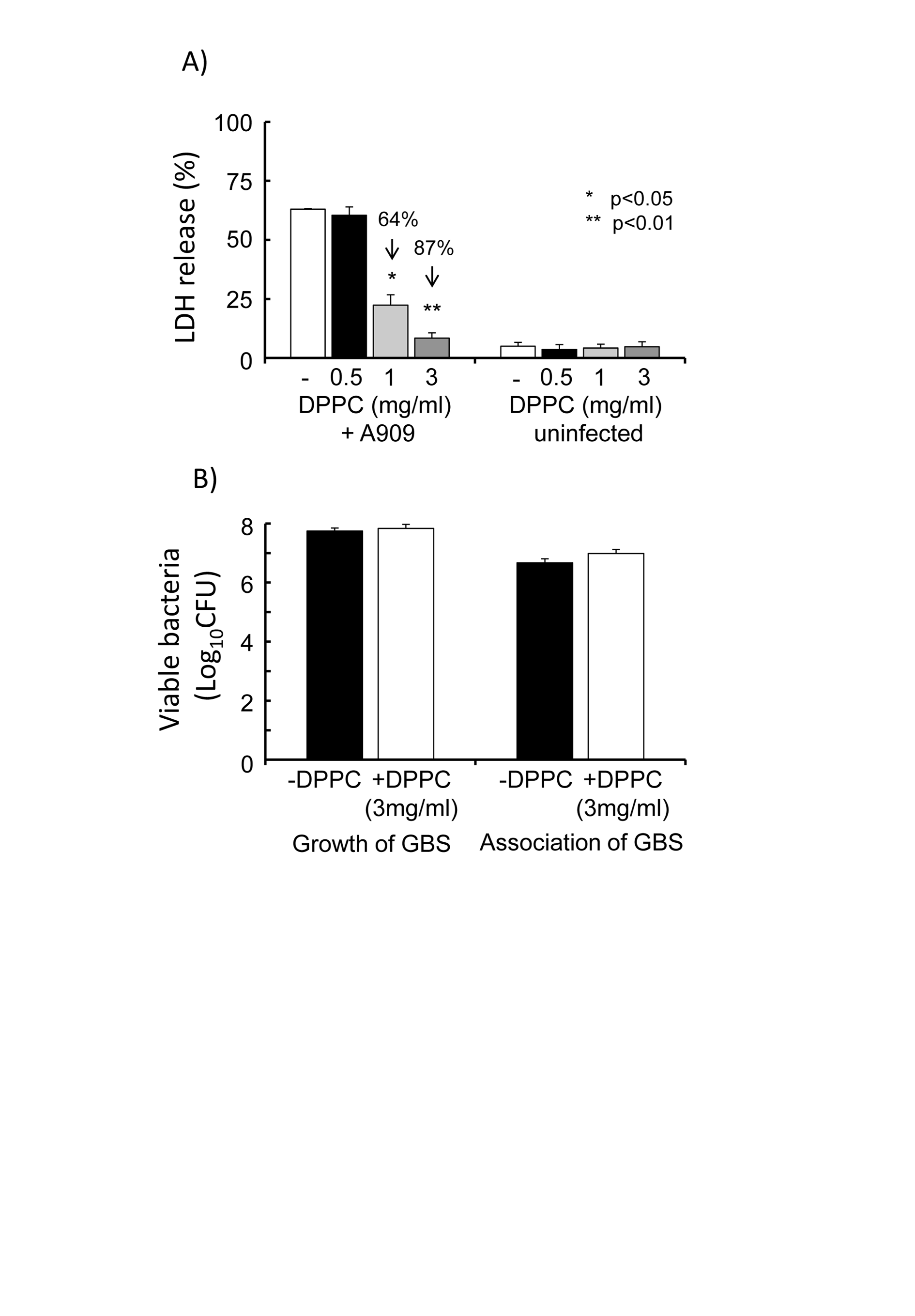

Supplement: Figure S4 — DPPC dose-dependent reduction of LDH release from GBS-infected meningioma cells. A) Various doses of DPPC were added to meningioma cells on challenge (t = 0 h) with GBS strain A909 (initial MOI 3000). LDH release was measured after 9 h. As controls, cells were infected without DPPC and were also maintained without infection in the presence of increasing amounts of DPPC. The columns show the mean level of LDH release and the error bars the SEM from n = 2 experiments. B) DPPC does not affect the growth of GBS or bacterial association with meningioma cells. Monolayers of meningioma cells were infected with strain NCTC10/84 in the presence or absence of DPPC (3 mg/ml) and bacterial growth in the medium and association with human cells was quantified after 9 h. The columns represent mean viable counts of bacteria and the error bars the SEM from at least 3 independent experiments. (TIF) [file pone.0042660.s004.tif]
