# Supplementary material for: Group B Streptococcus Interactions with Human Meningeal Cells and Astrocytes In Vitro
Source: PLoS One. 2012 Aug 10;7(8):e42660. doi: 10.1371/journal.pone.0042660 (PMC3416839; doi:10.1371/journal.pone.0042660)
Supplement: Table S1 — Infection of meningioma cells with GBS does not induce cytokine secretion. Meningioma cell lines (n = 2) were infected with various MOI of the GBS strains and cytokine secretion measured by ELISA after 24 h. As a control, cells were also infected with Neisseria meningitidis strain MC58 and wells were also left with medium alone (uninfected). The data are the mean levels of cytokine secretion (ng/ml) with the standard error of the mean (SEM in parenthesis) from n = 3 independent experiments. (DOCX) [file pone.0042660.s005.docx]

|  | | | | Mean Cytokine secretion (ng/ml) (± SEM) | | | |
| --- | --- | --- | --- | --- | --- | --- | --- |
| Treatment | | | MOI | IL-6 | IL-8 | MCP-1 | RANTES |
|  | |  |  |  |  |  |  |
| GBS strain | | H36B | 0.0003 | 0.22 (0.13) | 2.4 (0.2) | 0.22 (0.2) | 0.25 (0.25) |
|  | |  | 0.003 | 0.26 (0.18) | 2.4 (0.1) | 0.16 (0.1) | 0.2 (0.2) |
|  | |  | 0.3 | 0.23 (0.12) | 2.3 (0.2) | 0.16 (0.1) | 0.2 (0.2) |
|  | |  | 30 | 0.20 (0.12) | 2.0 (0.8) | 0.23 (0.2) | 0.2 (0.2) |
| Medium | | - | - | 0.95 (0.71) | 4.65 (1.23) | 8.9 (1.76) | 0.5 (0.5) |
| MC58 | | *-* | 0.3 | 903 (720) | 285 (125) | 119 (51) | 17 (6.3) |
|  | |  |  |  |  |  |  |
| GBS strain | | 18RS21 | 0.0003 | 0.08 (0.02) | 3.2 (1.4) | 0.28 (0.2) | 0.22 (0.22) |
|  | |  | 0.03 | 0.05 (0.02) | 2.2 (0.5) | 0.19 (0.19) | 0.2 (0.2) |
|  | |  | 0.3 | 0.07 (0.03) | 2.1 (0.5) | 0.21 (0.21) | 0.1 (0.1) |
|  | |  | 30 | 0.08 (0.03) | 2.3 (0.9) | 0.26 (0.26) | 0.2 (0.2) |
| Medium | | - | - | 0.11 (0.04) | 2.5 (0.8) | 5.3 (2.1) | 0.54 (0.5) |
| MC58 | | *-* | 0.3 | 98 (39) | 249 (90) | 83 (35) | 20 (7.2) |
|  | |  |  |  |  |  |  |
| GBS strain | | NEM316 | 0.0003 | 0.04 (0.03) | 2.2 (1.5) | 0.4 (0.3) | 0.3 (0.3) |
|  | |  | 0.003 | 0.03 (0.02) | 2.3 (1.4) | 0.38 (0.38) | 0.2 (0.2) |
|  | |  | 0.3 | 0.06 (0.04) | 2.4 (1.3) | 0.33 (0.33) | 0.2 (0.2) |
|  | |  | 30 | 0.06 (0.04) | 2.8 (1.2) | 0.3 (0.3) | 0.1 (0.1) |
| Medium | | - | - | 0.07 (0.02) | 2.7 (1.9) | 6.9 (3.3) | 1.0 (1.0) |
| MC58 | | *-* | 0.3 | 115 (78) | 157 (51) | 44 (7.2) | 22 (3.4) |
|  | |  |  |  |  |  |  |
| GBS strain | | 2603V/R | 0.0003 | 0.03 (0.02) | 1.7 (1.1) | 0.22 (0.2) | 0.1 (0.1) |
|  | |  | 0.003 | 0.01 (0.01) | 1.5 (0.9) | 0.15 (0.1) | 0.2 (0.2) |
|  | |  | 0.3 | 0.01 (0.01) | 1.2 (0.7) | 0.15 (0.14) | 0.1 (0.1) |
|  | |  | 30 | 0.02 (0.01) | 1.2 (0.8) | 0.19 (0.19) | 0.1 (0.1) |
| Medium | | - | - | 0.05 (0.02) | 1.8 (0.9) | 4.3 (2.4) | 0.25 (0.23) |
| MC58 | | - | 30 | 39 (15) | 175 (53) | 58 (23) | 12 (5) |
|  | |  |  |  |  |  |  |
| GBS strain | | COH-1 | 0.0003 | 0.03 (0.01) | 0.34 (0.11) | 0.04 (0.00) | 0.06 (0.01) |
|  | |  | 0.003 | 0.03 (0.00) | 0.49 (0.04) | 0.05 (0.02) | 0.03 (0.01) |
|  | |  | 0.3 | 0.03 (0.00) | 0.43 (0.16) | 1.78 (1.73) | 0.07 (0.05) |
|  | |  | 30 | 0.04 (0.01) | 0.48 (0.12) | 4.62 (0.1) | 0.04 (0.04) |
| Medium | | - | - | 0.04 (0.00) | 0.12 (0.02) | 3.86 (0.82) | 0.07 (0.01) |
| MC58 | | *-* | 0.3 | 3.8 (0.6) | 83 (11) | 25 (3.7) | 41 (0.8) |
|  | |  |  |  |  |  |  |
| GBS strain | | HY106 | 0.0003 | 0.05 (0.01) | 1.12 (0.01) | 0.82 (0.67) | 0.08 (0.01) |
|  | |  | 0.003 | 0.02 (0.01) | 0.98 (0.19) | 3.83 (0.43) | 0.14 (0.06) |
|  | |  | 0.3 | 0.03 (0.00) | 1.1 (0.37) | 2.71 (1.21) | 0.09 (0.02) |
|  | |  | 30 | 0.04 (0.00) | 0.87 (0.14) | 5.3 (0.3) | 0.13 (0.03) |
| Medium | | - | - | 0.04 (0.00) | 0.12 (0.02) | 3.86 (0.82) | 0.07 (0.01) |
| MC58 | | - | 30 | 3.8 (0.6) | 83 (11) | 25 (3.7) | 41 (0.8) |
|  | | |  |  |  |  |  |
| GBS strain | | A909 (24h) | 0.0003 | 0.04 (0.04) | 2.8 (0.6) | 0.06 (0.06) | 0.06 (0.06) |
|  | |  | 0.003 | 0.05 (0.05) | 3.1 (0.6) | 0.1 (0.1) | 0.02 (0.01) |
|  | |  | 0.3 | 0.05 (0.05) | 3.1 (0.4) | 0.17 (0.17) | 0.03 (0.03) |
|  | |  | 30 | 0.08 (0.08) | 2.9 (0.4) | 0.24 (0.24) | 0.01 (0.01) |
| Medium | | - | - | 0.05 (0.04) | 2.2 (0.5) | 4.1 (1.5) | 0.16 (0.09) |
| MC58 | | - | 30 | 137 (85) | 187 (91) | 55 (24) | 14.6 (10.1) |
|  | |  |  |  |  |  |  |
| GBS strain | | A909 | 0.0003 | 0.07 (0.07) | 2.6 (1.1) | 4.7 (1.6) | 0.18 (0.1) |
|  | | Δ*cylE* (24h) | 0.003 | 0.02 (0.01) | 2.3 (0.9) | 4.4 (1.2) | 0.18 (0.1) |
|  | |  | 0.3 | 0.06 (0.06) | 2.6 (0.8) | 4.7 (0.09) | 0.21 (0.09) |
|  | |  | 30 | 0.05 (0.05) | 2.7 (0.6) | 5.2 (0.6) | 0.18 (0.04) |
| Medium | | - | - | 0.05 (0.04) | 2.4 (1.2) | 4.08 (1.5) | 0.13 (0.08) |
| MC58 | | - | 30 | 63 (34) | 145 (109) | 51 (25) | 18.1 (9.1) |
|  | |  |  |  |  |  |  |
| GBS | | A909 | 0.0003 | 0.14 (0.14) | 5.1 (0.9) | 3.6 (3.5) | 0.3 (0.15) |
| strain | | Δ*cylE* (48h) | 0.003 | 0.11 (0.11) | 4.8 (0.7) | 3.45 (3.45) | 0.3 (0.17) |
|  | |  | 0.3 | 0.06 (0.06) | 4.2 (0.1) | 3.17 (3) | 0.4 (0.3) |
|  | |  | 30 | 0.12 (0.12) | 4.5 (0.2) | 3.1 (3) | 0.3 (0.16) |
| Medium | | - | - | 0.07 (0.06) | 2.4 (1.5) | 4.8 (2.4) | 0.2 (0.16) |
| MC58 | | - | 30 | 247 (148) | 334 (72) | 105 (46) | 85 (19) |
|  | | |  |  |  |  |  |
| GBS | NCTC10/84 | | 0.0003 | 0.03 (0.01) | 0.01 (0.01) | 0.02 (0.01) | 0.0 |
| strain | (24h) | | 0.003 | 0.02 (0.01) | 0.01 (0.01) | 0.02 (0.00) | 0.0 |
|  |  | | 0.3 | 0.02 (0.0) | 0.01 (0.01) | 0.03 (0.01) | 0.0 |
|  |  | | 30 | 0.06 (0.04) | 0.01 (0.01) | 0.04 (0.00) | 0.0 |
| Medium | - | | - | 0.08 (0.02) | 0.03 (0.02) | 0.94 (0.86) | 0.03 (0.02) |
| MC58 | - | | 30 | 20 (2) | 290 (5) | 41.4 (33.6) | 186 (133) |
|  |  | |  |  |  |  |  |
| GBS | NCTC10/84 | | 0.0003 | 0.09 (0.07) | 0.01 (0.01) | 0.03 (0.01) | 0.0 |
| strain | Δ*cylE* (24h) | | 0.003 | 0.08 (0.06) | 0.01 (0.01) | 0.03 (0.01) | 0.0 |
|  |  | | 0.3 | 0.06 (0.03) | 0.03 (0.03) | 0.06 (0.03) | 0.0 |
|  |  | | 30 | 0.04 (0.02) | 0.02 (0.02) | 0.04 (0.01) | 0.0 |
| Medium |  | | - | 0.1 (0.08) | 0.03 (0.02) | 0.94 (0.86) | 0.03 (0.02) |
| MC58 |  | | 30 | 11.3 (9.7) | 292 (3) | 37.3 (2.9) | 186 (133) |
|  |  | |  |  |  |  |  |
| GBS | NCTC10/84 | | 0.0003 | 0.1 (0.07) | 0.01 (0.01) | 0.01 (0.00) | 0.0 |
| strain | Δ*cylE* (48h) | | 0.003 | 0.08 (0.06) | 0.02 (0.00) | 0.01 (0.00) | 0.02 (0.02) |
|  |  | | 0.3 | 0.06 (0.04) | 0.09 (0.06) | 0.01 (0.00) | 0.05 (0.05) |
|  |  | | 30 | 0.04 (0.02) | 0.04 (0.01) | 0.02 (0.01) | 0.01 (0.01) |
| Medium |  | | - | 0.1 (0.08) | 0.05 (0.01) | 0.39 (0.00) | 0.1 (0.01) |
| MC58 |  | | 30 | 30 (28) | 1092 (975) | 55.3 (3.6) | 272 (38) |
