# Supplementary material for: Group B Streptococcus Interactions with Human Meningeal Cells and Astrocytes In Vitro
Source: PLoS One. 2012 Aug 10;7(8):e42660. doi: 10.1371/journal.pone.0042660 (PMC3416839; doi:10.1371/journal.pone.0042660)
Supplement: Table S2 — Infection of meningioma cells by heat-killed GBS does not induce cytokine secretion. Meningioma cells were infected with heat-killed GBS strain NEM316 and cytokine production measured after 24 and 48 h. Data are the mean levels of cytokine secretion (ng/ml) with standard deviation (SD) of n = 3 wells and they are similar to data presented for viable bacteria in Table S1 for NEM316. Heat-killed MC58 bacteria show a reduced capacity to induce cytokines compared to viable bacteria (Table S1). (DOCX) [file pone.0042660.s006.docx]

|  | | | Mean Cytokine secretion (ng/ml) (± SD) | | | |
| --- | --- | --- | --- | --- | --- | --- |
| Heat-killed treatment | | MOI | IL-6 | IL-8 | MCP-1 | RANTES |
|  |  |  |  |  |  |  |
| GBS strain | NEM316 | 0.0003 | 0.13 (0.00) | 0.53 (0.39) | 1.43 (0.85) | 2.56 (0.33) |
|  | (24h) | 0.003 | 0.14 (0.05) | 0.44 (0.23) | 1.39 (0.46) | 2.45 (0.02) |
|  |  | 0.3 | 0.12 (0.04) | 0.46 (0.2) | 1.41 (0.38) | 2.17 (0.17) |
|  |  | 30 | 0.33 (0.01) | 0.96 (0.06) | 1.68 (0.16) | 2.33 (0.25) |
| Medium | - | - | 0.06 (0.01) | 0.28 (0.03) | 0.89 (0.09) | 2.04 (0.13) |
|  |  |  |  |  |  |  |
| GBS strain | NEM316 | 0.0003 | 0.20 (0.01) | 0.35 (0.04) | 1.26 (0.2) | 0.54 (0.53) |
|  | (48h) | 0.03 | 0.19 (0.03) | 0.30 (0.03) | 1.10 (0.04) | 0.69 (0.03) |
|  |  | 0.3 | 0.15 (0.03) | 0.31 (0.05) | 1.15 (0.16) | 0.16 (0.22) |
|  |  | 30 | 0.28 (0.07) | 0.75 (0.09) | 1.96 (0.17) | 0.81 (0.70) |
| Medium | - | - | 0.18 (0.02) | 0.38 (0.04) | 1.27 (0.16) | 0.22 (0.21) |
| MC58 | *-* | 0.3 | 2.89 (0.81) | 8.04 (0.38) | 8.30 (0.24) | 8.69 (0.68) |
