# Supplementary material for: Group B Streptococcus Interactions with Human Meningeal Cells and Astrocytes In Vitro
Source: PLoS One. 2012 Aug 10;7(8):e42660. doi: 10.1371/journal.pone.0042660 (PMC3416839; doi:10.1371/journal.pone.0042660)
Supplement: Table S3 — Infection of SVGmm astrocytes with GBS does not induce cytokine secretion. Astrocyte cell lines (n = 3 experiments) were infected with various MOI of the GBS strains A909 and A909ΔcylE and cytokine secretion measured by ELISA after 24 h. As a control, cells were also infected with Neisseria meningitidis strain MC58 and wells were also left with medium alone (uninfected). The data are the mean levels of cytokine secretion (ng/ml) with the standard deviation (in parenthesis) of triplicate wells from a representative experiment. (DOCX) [file pone.0042660.s007.docx]

|  | | | Mean Cytokine secretion (ng/ml) (± SD) | | | |
| --- | --- | --- | --- | --- | --- | --- |
| Treatment | | MOI | IL-6 | IL-8 | MCP-1 | RANTES |
| GBS strain | A909 | 0.0003 | 0.76 (0.04) | 1.19 (0.00) | 2.16 (0.19) | 0.17 (0.15) |
|  |  | 0.003 | 0.76 (0.01) | 1.16 (0.01) | 2.38 (0.36) | 0.26 (0.24) |
|  |  | 0.3 | 0.76 (0.01) | 1.18 (0.01) | 2.22 (0.22) | 0.00 (0.00) |
|  |  | 30 | 0.77 (0.01) | 1.18 (0.01) | 1.76 (0.12) | 0.32 (0.21) |
|  |  |  |  |  |  |  |
| GBS strain | A909Δ*cylE* | 0.0003 | 0.76 (0.01) | 1.12 (0.00) | 1.71 (0.28) | 0.46 (0.54) |
|  |  | 0.003 | 0.75 (0.01) | 1.15 (0.01) | 1.79 (0.25) | 0.56 (0.64) |
|  |  | 0.3 | 0.76 (0.01) | 1.16 (0.00) | 1.93 (0.21) | 0.00 (0.00) |
|  |  | 30 | 0.77 (0.00) | 1.15 (0.04) | 1.82 (0.19) | 0.21 (0.3) |
| Medium | - | - | 0.74 (0.04) | 1.18 (0.01) | 1.49 (0.11) | 0.42 (0.6) |
| MC58 | - | 0.3 | 2.44 (1.26) | 4.07 (0.54) | 4.65 (2.62) | 0.49 (0.42) |
|  |  |  |  |  |  |  |
